# Supplementary figures and images for: A novel peptidoglycan deacetylase modulates daughter cell separation in E. coli
Source: PLoS Genet. 2025 Sep 5;21(9):e1011626. doi: 10.1371/journal.pgen.1011626 (PMC12440217; doi:10.1371/journal.pgen.1011626)

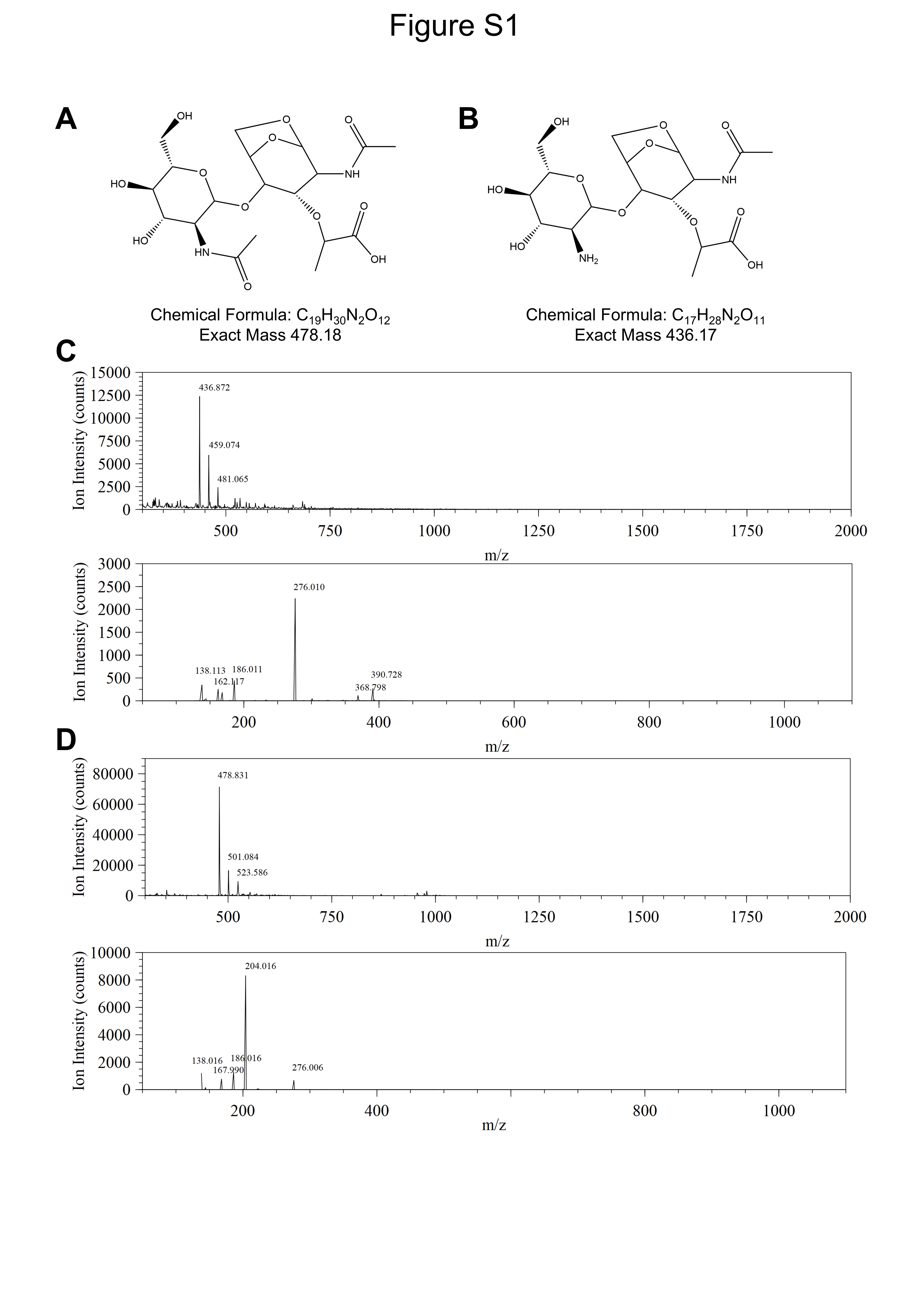

Supplement: S1 Fig — (A, B) Chemical structures of GlcNAc-MurNAcAnh and GlcN-MurNAcAnh, respectively. (C) and (D), verification of the identity of HPLC peaks labelled in Fig. 1G as GlcN-MurNAcAnh and GlcNAc-MurNAcAnh, respectively, by MS and MS/MS. MS/MS analysis of peaks from LC-MS analysis from PG digested with MltA resulted in the same fragmentation shown here. (C) Verification using MS/MS (bottom) that the peak with m/z 437 (top) corresponds to GlcN-MurNAcAnh. Fragmentation of GlcN-MurNAcAnh yields GlcN(-H2O)+H+ (m/z 162), and not GlcNAc(-H2O)+H+ (m/z 204), and MurNAcAnh + H+ (m/z 276). (D) Verification using MS/MS (bottom) that peak with m/z 479 (top) corresponds to GlcNAc-MurNAcAnh. Fragmentation of GlcNAc-MurNAcAnh yields the GlcNAc(-H2O)+H+ ion (m/z 204) and MurNAcAnh + H+ (m/z 276). (TIF) [file pgen.1011626.s001.tif]

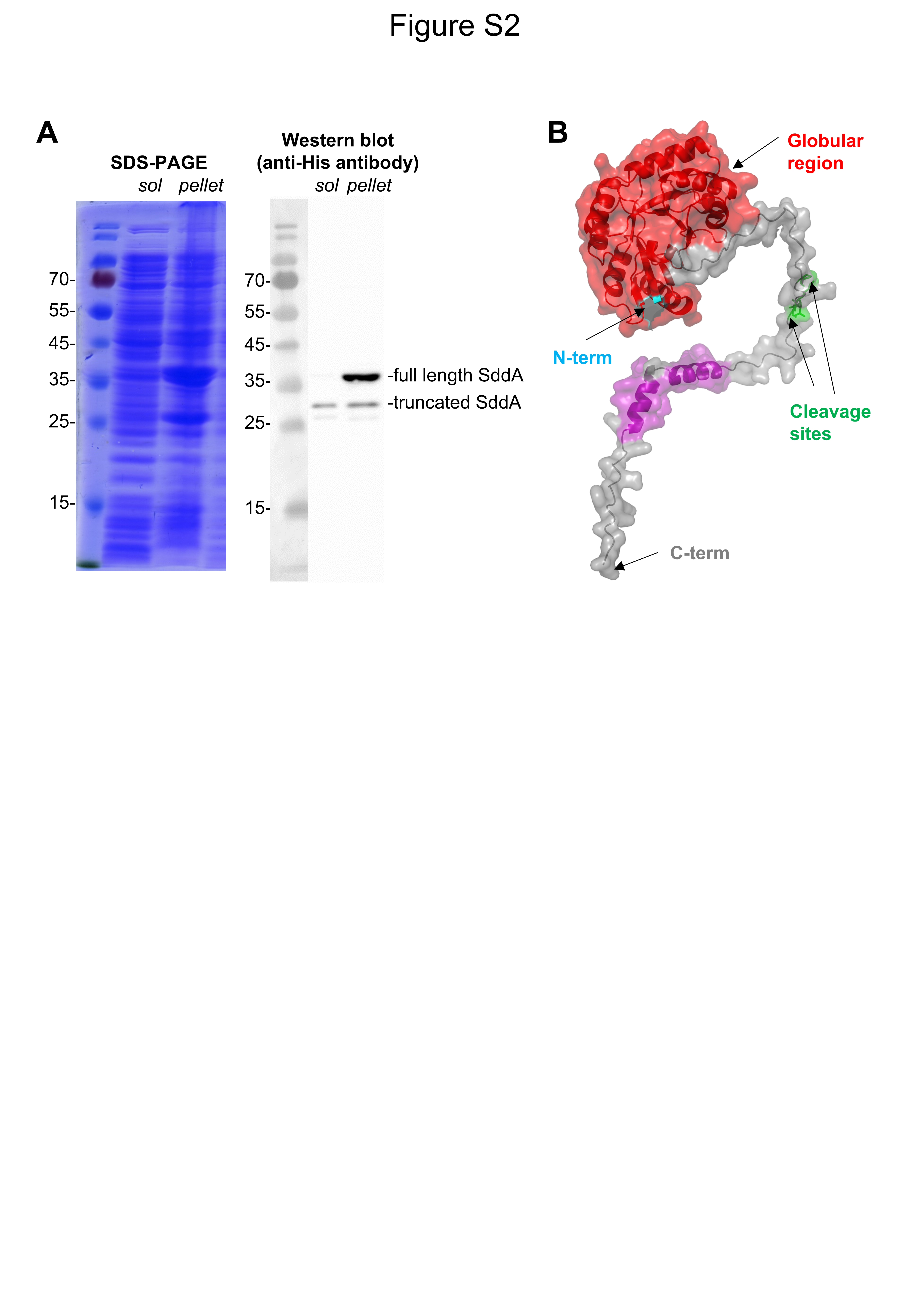

Supplement: S2 Fig — (A) SDS-PAGE and Western-Blot analysis of soluble (sol) and pellet fractions from sonicated cell extracts of BL21(DE3) pET28a-HisSddA induced with IPTG. The His-tag in the N-terminus His-SddA was detected using anti-His-tag antibody. (B) AlphaFold model of E. coli SddA showing the predicted globular (red) and unfolded C-terminal (grey) regions, plus the location of cleavage sites in His-SddA purified from soluble extracts as the one analysed in A. The identified cleavage sites are P258, V262, K263 and L264. (TIF) [file pgen.1011626.s002.tif]

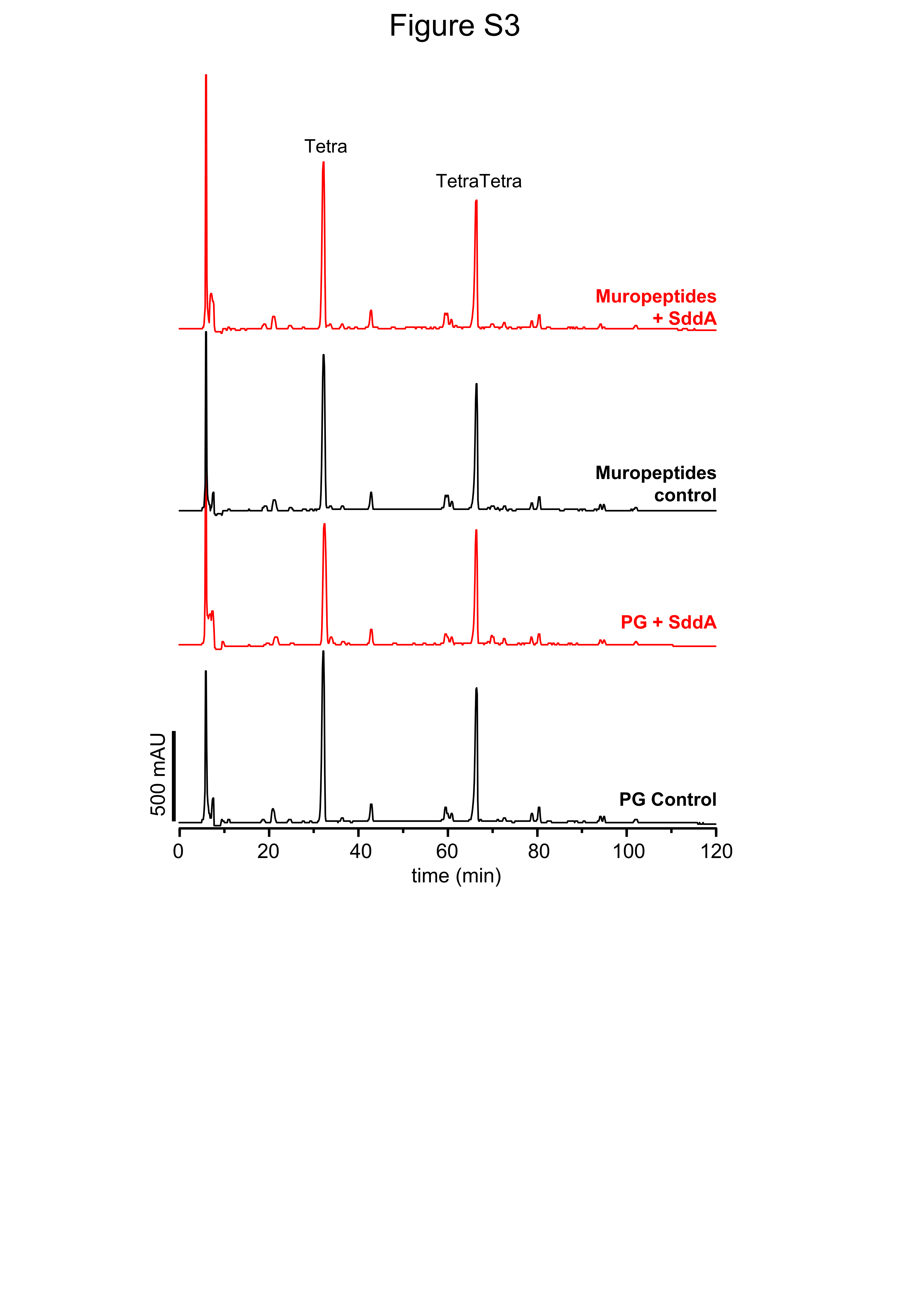

Supplement: S3 Fig — PG was incubated first with SddA or buffer and then with the muramidase cellosyl (chromatograms labelled “PG”) or first with cellosyl and then with SddA or buffer (chromatograms labelled “muropeptides”), to test the activity of SddA on PG or muropeptides, respectively. SddA was added at 10 µM in both cases. SddA treatment did not introduce any changes in the resulting chromatograms. (TIF) [file pgen.1011626.s003.tif]

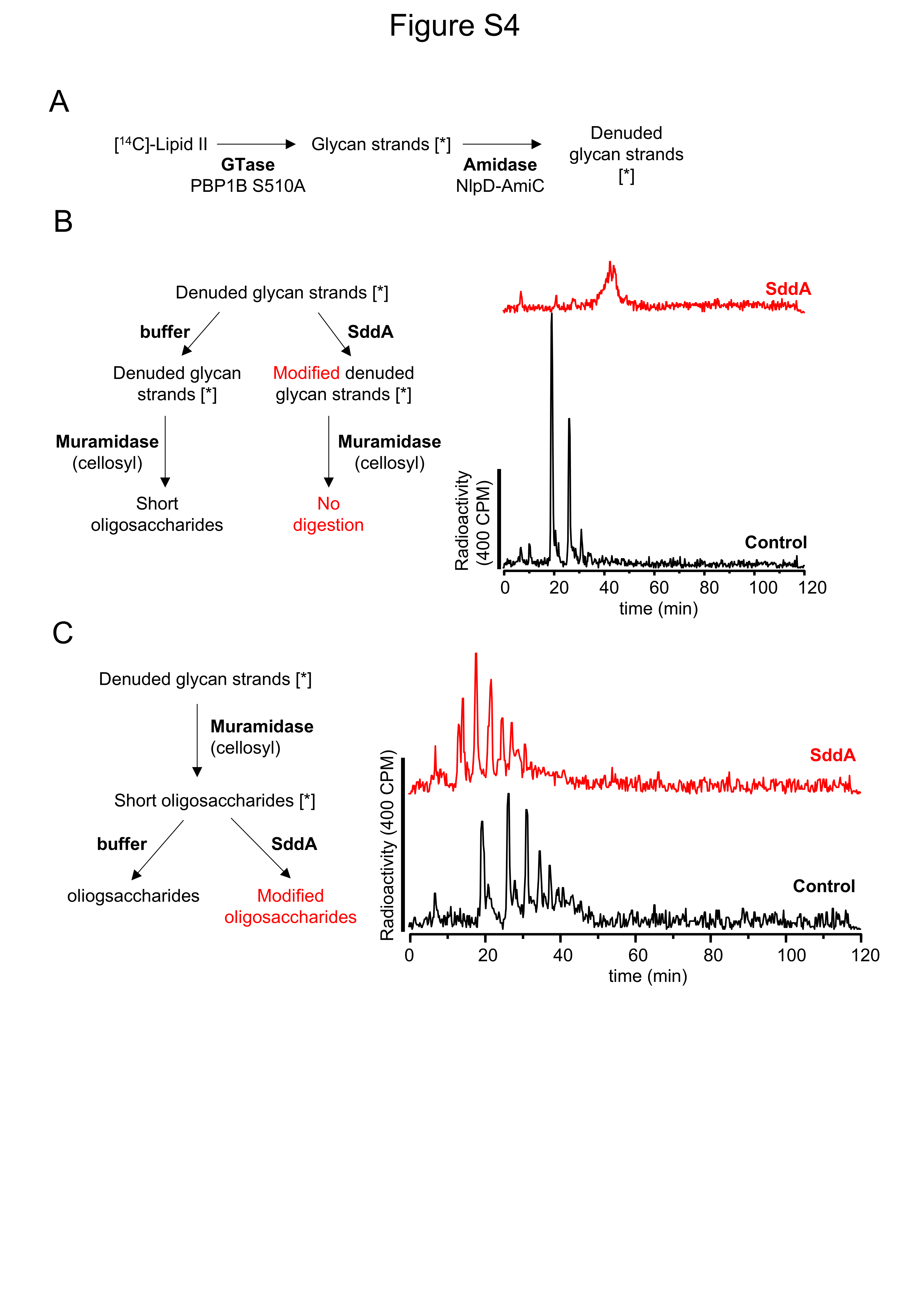

Supplement: S4 Fig — (A) Radiolabelled denuded glycan strands were prepared from radiolabelled lipid II ([14C]-lipid II) using transpeptidase-defective PBP1B (S510A), and the amidase AmiC plus its activator NlpD. (B) Scheme depicting the preparation of samples (left side) and their analysis (chromatograms on the right side). Muramidase was unable to digest the denuded glycan strands treated with SddA. (C) Scheme depicting the preparation of samples (left side) and their analysis (chromatograms on the right side). SddA modified the short oligosaccharides obtained by digesting radiolabelled denuded glycan strands with a muramidase. (TIF) [file pgen.1011626.s004.tif]

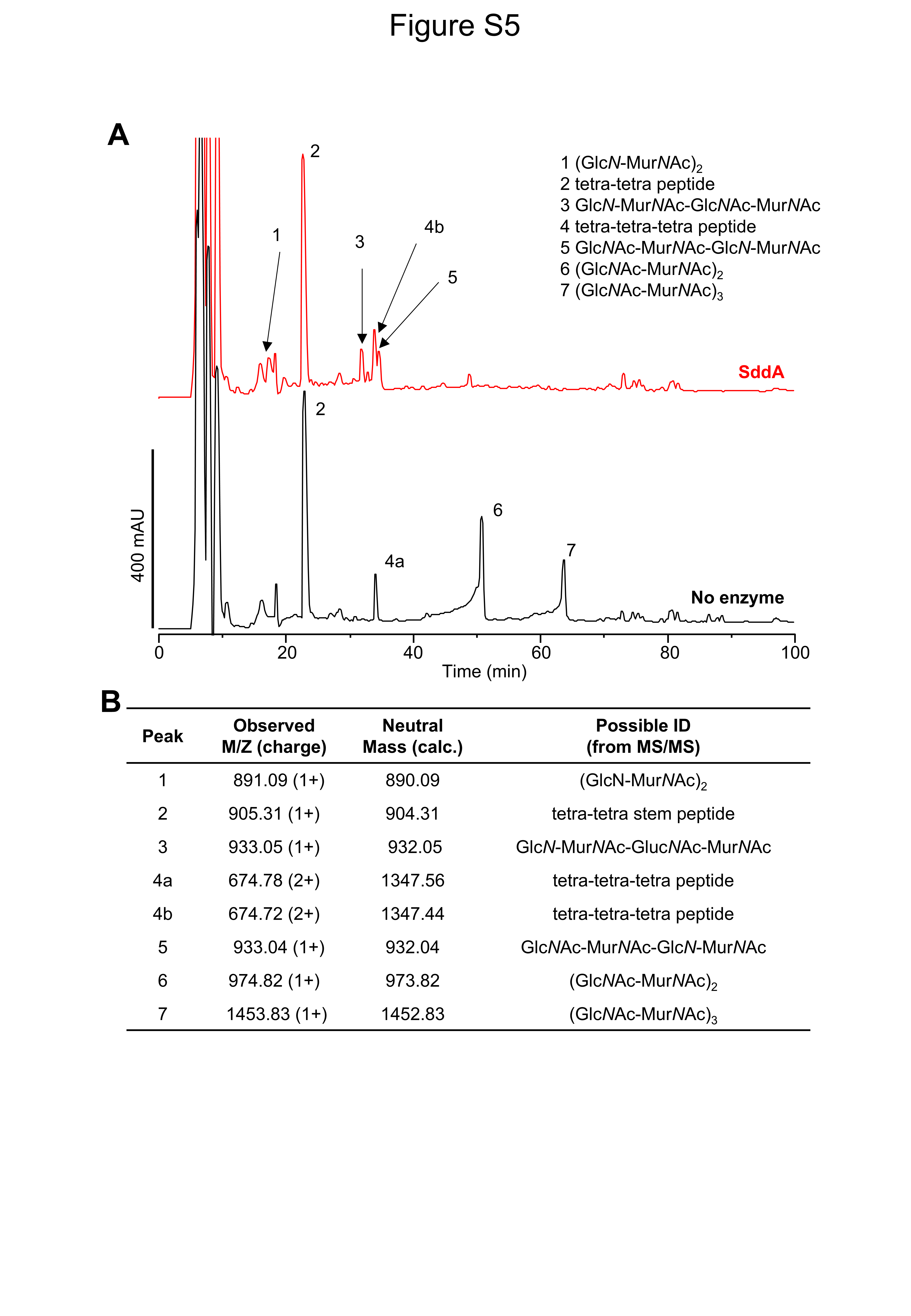

Supplement: S5 Fig — (A) Chromatograms of muramidase-digested denuded glycan strands, treated with SddA (red) or buffer (black) after digestion with the muramidase cellosyl. (B) Results of MS and MS/MS analysis of the labelled peaks in A. All muropeptide peaks correspond to the non-reduced species. (TIF) [file pgen.1011626.s005.tif]

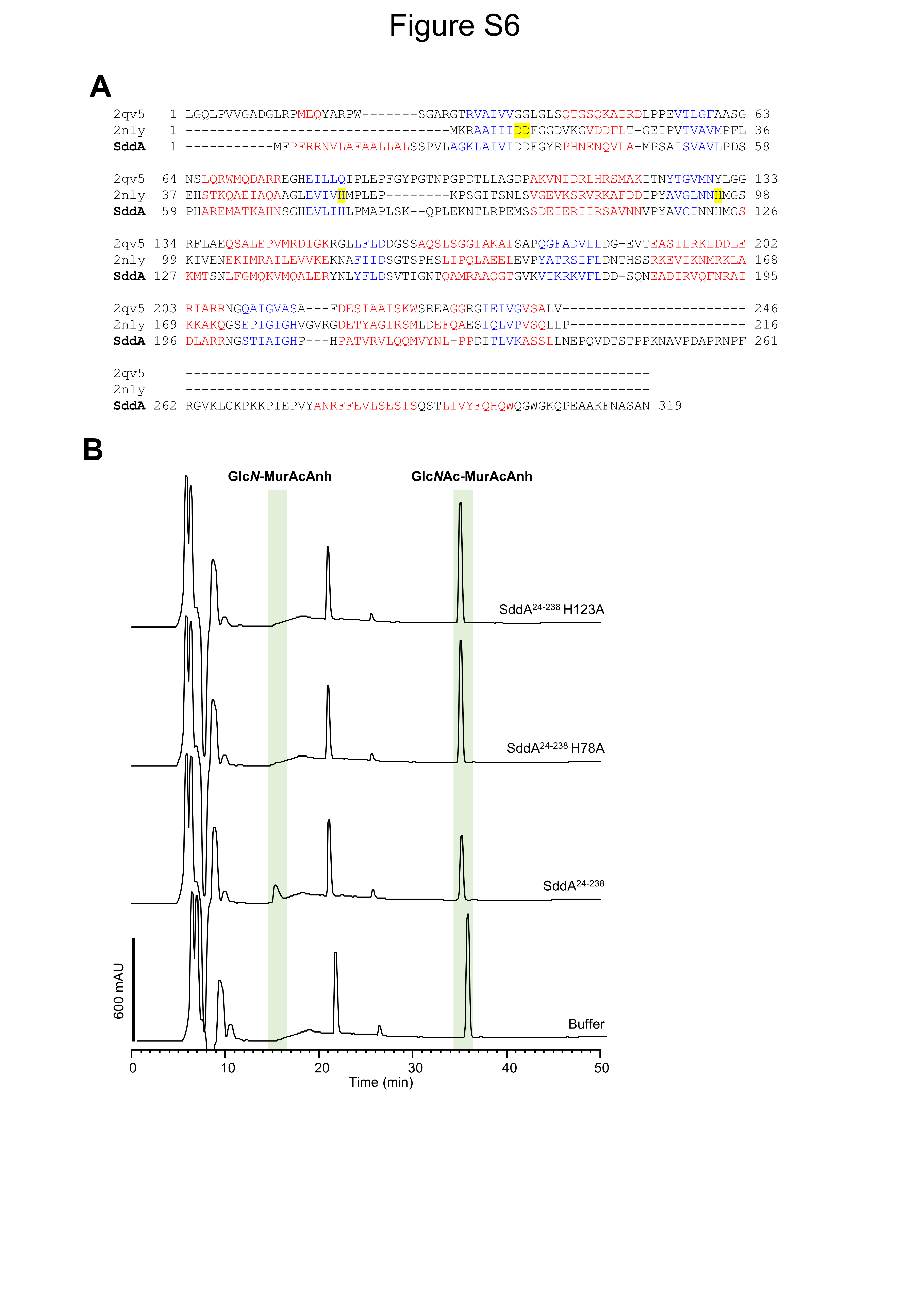

Supplement: S6 Fig — (A) Sequence alignment of E. coli SddA with the sequences of the two proteins with a divergent polysaccharide deacetylase domain (PF04748) whose crystal structure is available. These two proteins are BH1492 from Bacillus halodurans (PDB 2NLY), which contains a Zn2+ in the crystal structure, and ATU2773 from Agrobacterium tumefaciens (PDB 2QV5), which does not contain a Zn2+ ion in the crystal structure. The sequences are coloured by secondary structure (red indicating alpha helix and blue beta strand). The residues coordinating Zn2+ in BH1492 are highlighted in yellow. (B) Chromatograms of the analysis of denuded glycan strands treated first with buffer, SddA, SddA24-238, SddA24-238 H78A, or SddA24-238 H123A, and then with the lytic transglycosylase MltA to produce anhydro-muropeptides. Chromatograms for assays with H78A and H123A SddA variants show no GlcN-MurAcAnh peak, indicating they are inactive. (TIF) [file pgen.1011626.s006.tif]

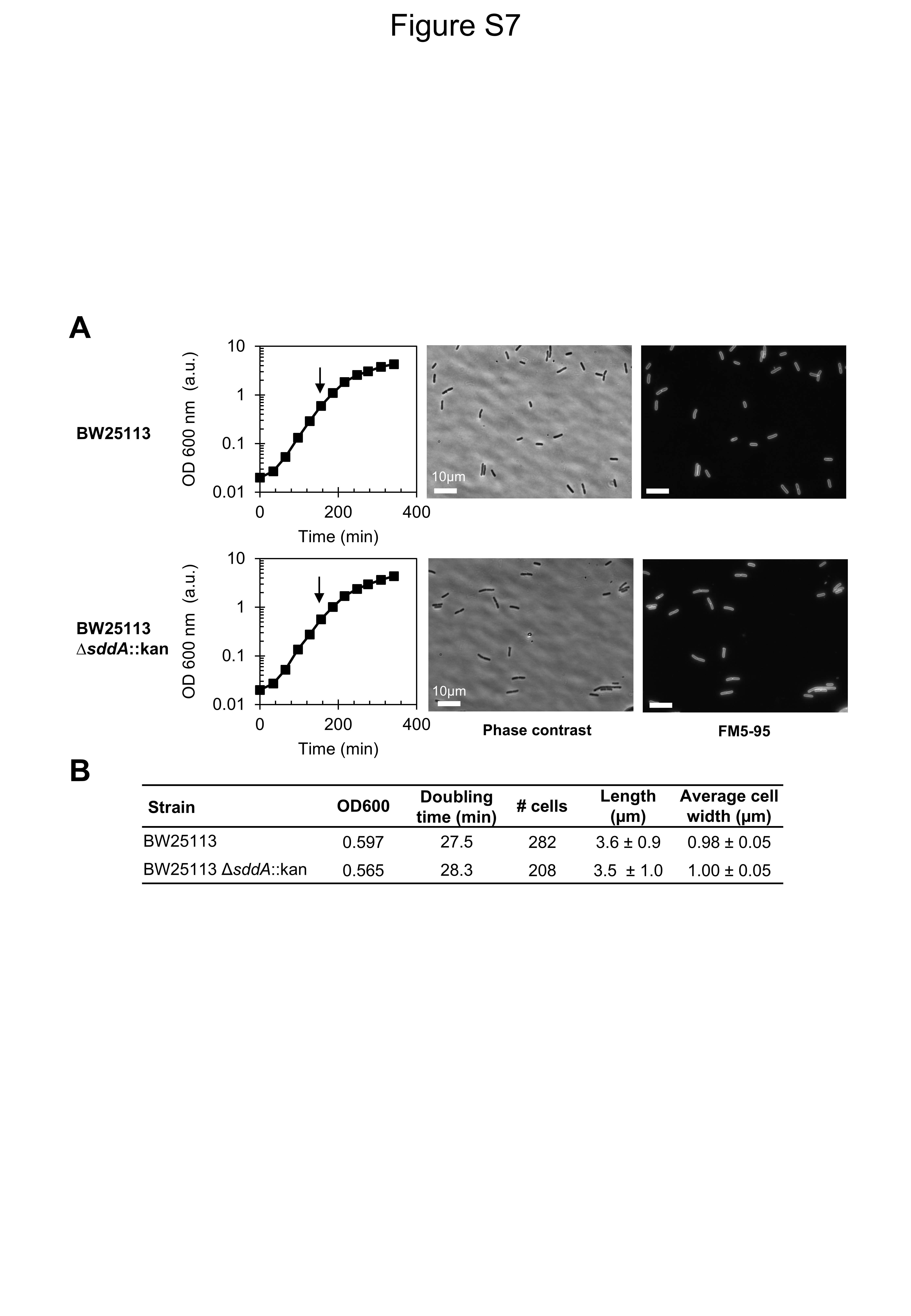

Supplement: S7 Fig — (A) BW25113 or BW25513 ΔssdA::kan were grown in LB at 37°C. Samples were collected (arrows), fixed, stained with FM5–95 (cell membrane), immobilized and imaged by phase contrast and epifluorescence microscopy. Representative images are shown. Scale bar is 10 µm. (B) morphological measurements and growth doubling time of the cells and growth curves shown in A. (TIF) [file pgen.1011626.s007.tif]

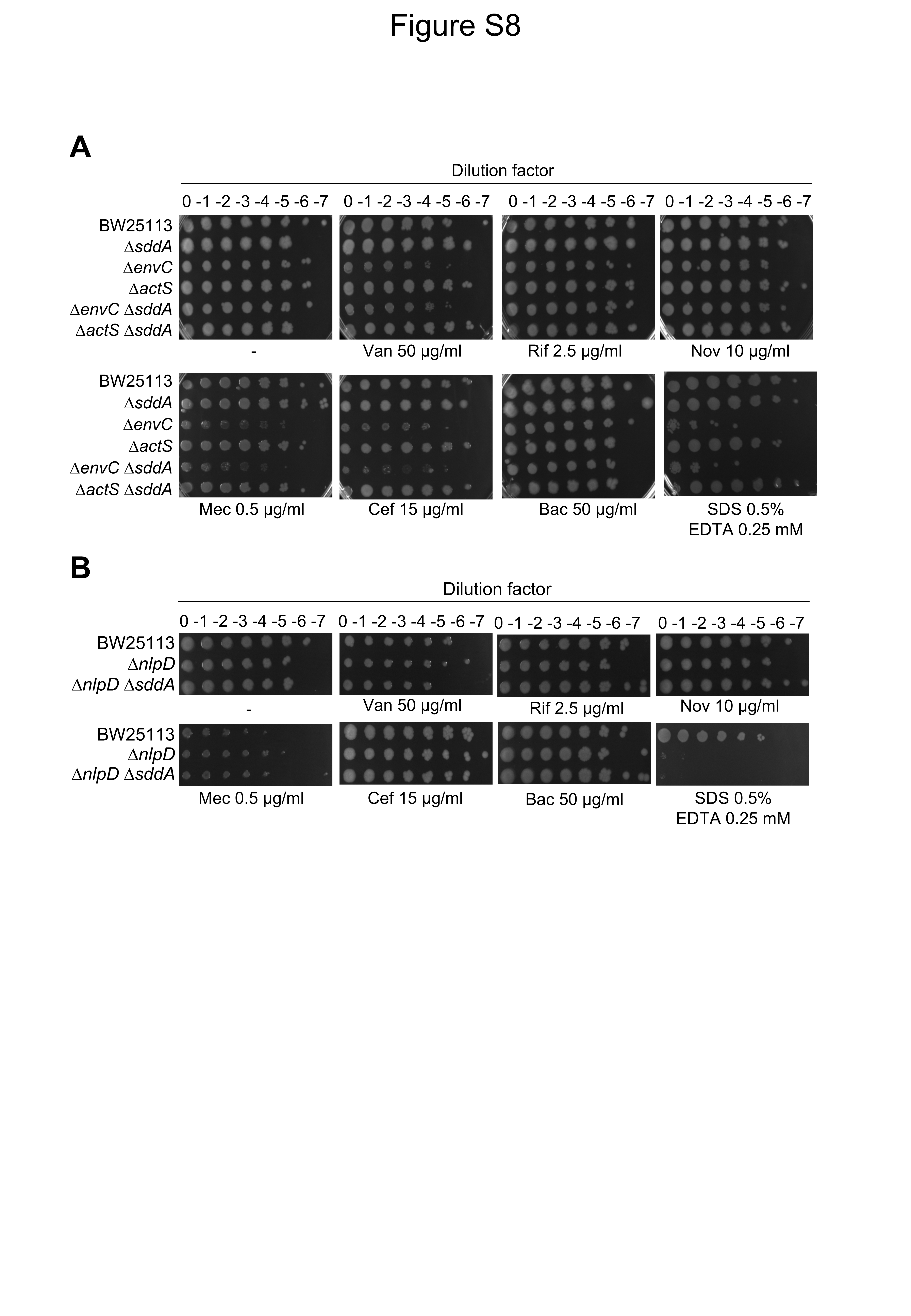

Supplement: S8 Fig — (A) Overnight cultures of BW25113 and isogenic ΔsddA, ΔenvC, ΔactS, ΔenvC ΔsddA, ΔactS ΔsddA mutants and (B) of BW25113 ΔnlpD and isogenic ΔnlpD ΔsddA were serially diluted and spotted on LB with 5% NaCl plates containing vancomycin (Van), rifampicin (Rif), novobiocin (Nov), mecillinam (Mec), cefsulodin (Cefs), bacitracin (Bac) or SDS/EDTA at the indicated concentrations. Plates were incubated at 37°C for 24 h. (TIF) [file pgen.1011626.s008.tif]

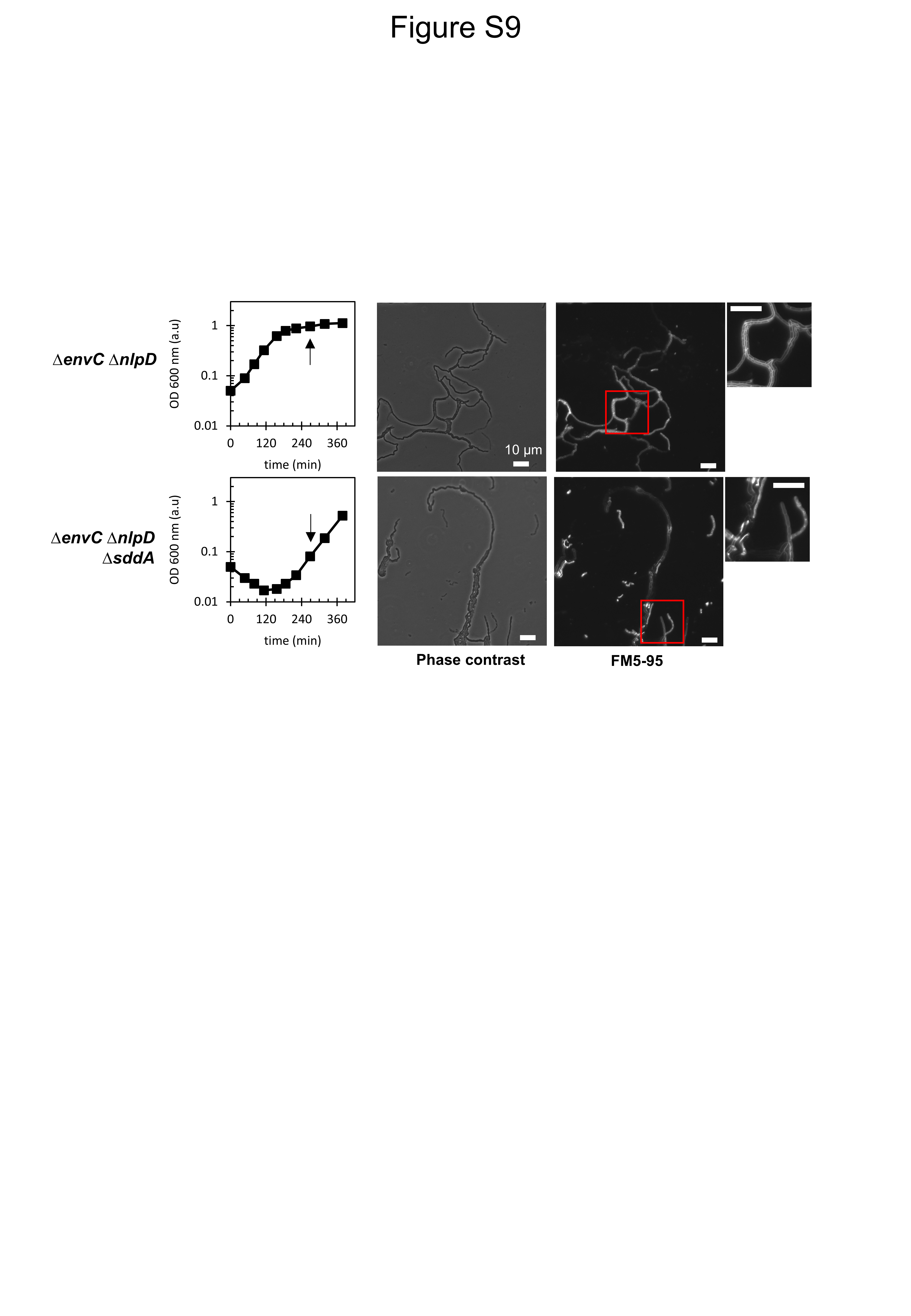

Supplement: S9 Fig — BW25113 ∆envC ∆nlpD and BW25113 ∆envC ∆nlpD ∆sddA were grown in LB at 37°C. At indicated times, samples were taken and imaged on agarose pads containing FM5–95 membrane stain. All scale bars represent 10 µM. (TIF) [file pgen.1011626.s009.tif]

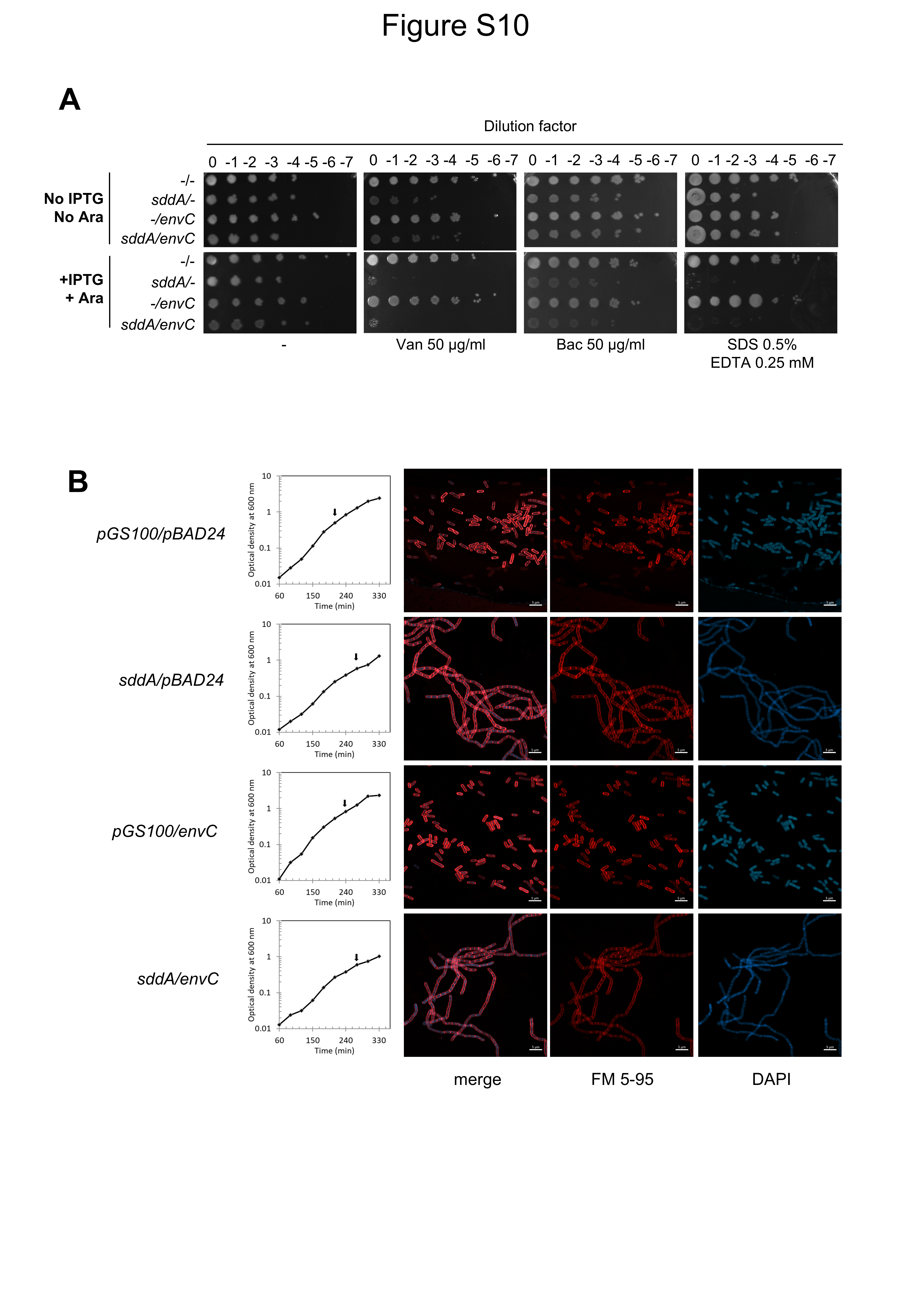

Supplement: S10 Fig — (A) Overnight cultures of BW25113 harbouring empty pGS100 or pBAD24 plasmids (-/-) or pGS100 with sddA (sddA/-) or pBAD24 with envC (-/envC) or both (ssdA/envC) were serially diluted and spotted onto LB-Lennox supplemented with 25 μg ml-1 chloramphenicol and 100 μg ml-1 ampicillin containing vancomycin (Van), bacitracin (Bac) or SDS/EDTA at the indicated concentrations. 0.2% arabinose and 0.5 mM IPTG were used to induce envC and sddA expression, respectively. (B) BW25113 cells harbouring pGS100 and pBAD24 or pBAD24 with envC (pGS100/envC), pGS100 with sddA (sddA/pBAD24) or both (ssdA/envC) were grown in LB with 5% NaCl supplemented with 25 μg ml-1 chloramphenicol and 100 μg ml-1 ampicillin. IPTG (0.5 mM) and arabinose (0.2%) were used to induce the expression of sddA and envC, respectively. Samples were collected at the exponential growth phase (arrows) stained with FM5–95 (red, cell membrane) and DAPI (blue, nucleoid), immobilized and imaged by confocal fluorescence microscopy. Representative images are shown. Scale bar is 5 µm. (TIF) [file pgen.1011626.s010.tif]

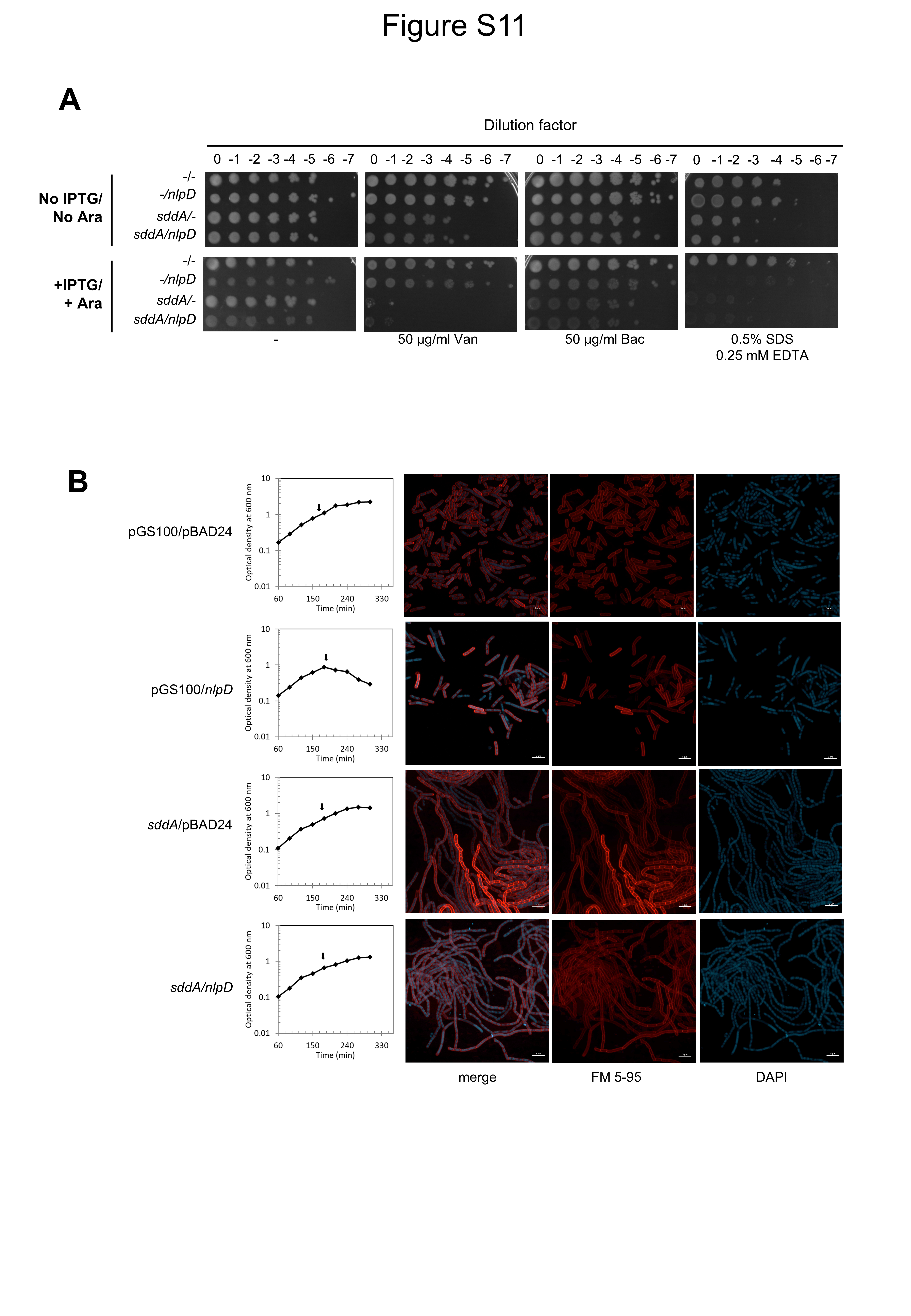

Supplement: S11 Fig — (A) Overnight cultures of BW25113 harbouring empty pGS100 or pBAD24 plasmids (-/-) or pGS100 with sddA (sddA/-) or pBAD24 with nlpD (-/nlpD) or both (ssdA/nlpD) were serially diluted and spotted onto LB-Lennox supplemented with 25 μg ml-1 chloramphenicol and 100 μg ml-1 ampicillin containing vancomycin (Van), bacitracin (Bac) or SDS/EDTA at the indicated concentrations. 0.2% arabinose and 0.5 mM IPTG were used to induce nlpD and sddA expression, respectively. (B) BW25113 cells harbouring pGS100 and pBAD24 or pBAD24 with nlpD (pGS100/nlpD), pGS100 with sddA (sddA/pBAD24) or both (ssdA/nlpD) were grown in LB-Lennox supplemented with 25 μg ml-1 chloramphenicol and 100 μg ml-1 ampicillin. IPTG (0.5 mM) and arabinose (0.2%) were used to induce the expression of sddA and nlpD, respectively. Samples were collected at the exponential growth phase (arrows) stained with FM5–95 (red, cell membrane) and DAPI (blue, nucleoid), immobilized and imaged by confocal fluorescence microscopy. Representative images are shown. Scale bar is 5 µm. (TIF) [file pgen.1011626.s011.tif]

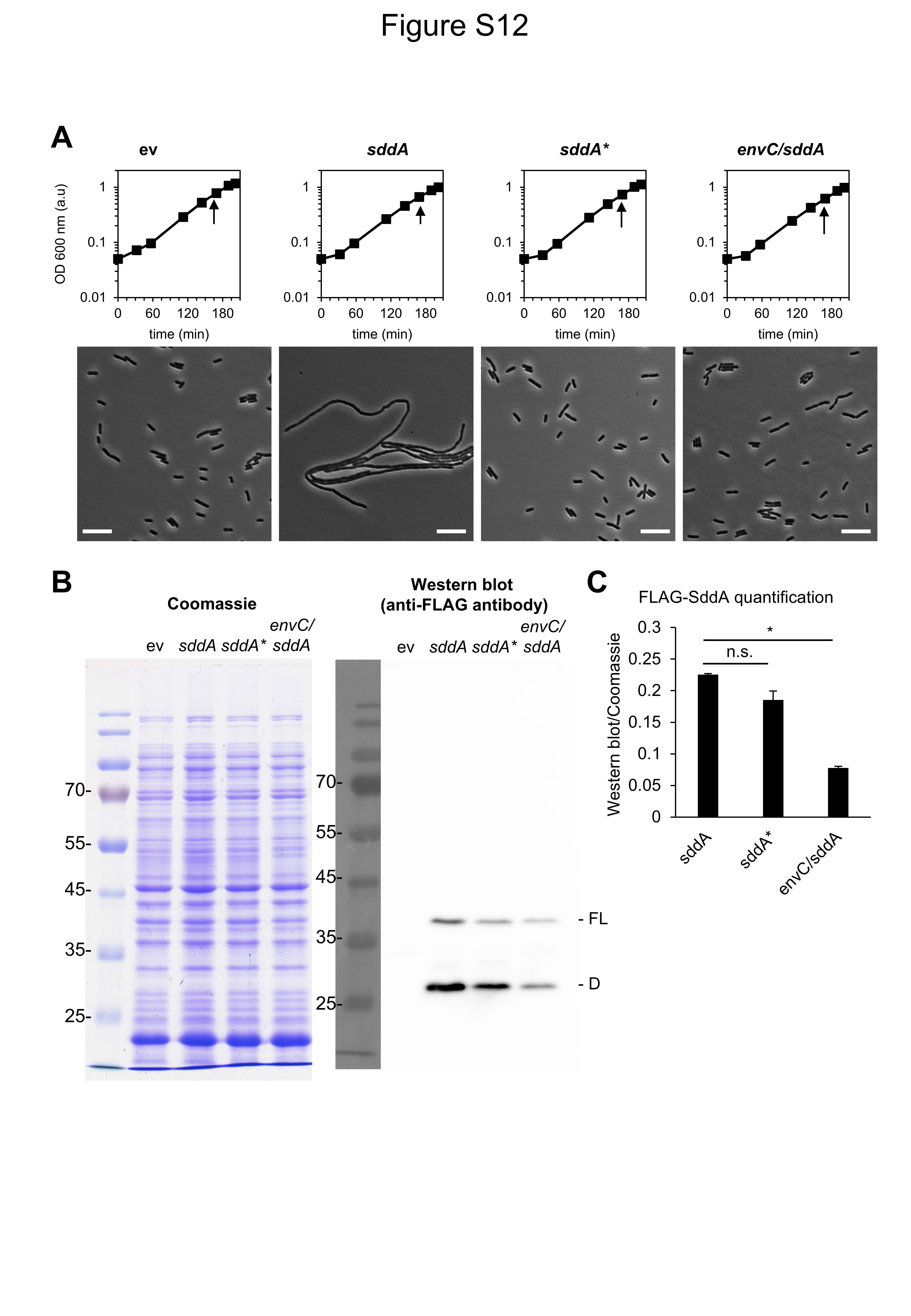

Supplement: S12 Fig — (A) BW25113 cells harbouring pGS100 or pGS100 encoding FLAG-sddA (sddA), FLAG-sddA H123A (sddA*) or envC/FLAG-sddA (envC/sddA) were grown in LB supplemented with 25 μg ml-1 chloramphenicol. 0.5 mM IPTG was added at an OD600 of 0.1, and sample for microscopy were taken when indicated in the growth curves. Cells were imaged using phase contrast. Scale bar is 10 µm. (B) BW25113 cells harbouring the same plasmids as described in (A) and grown in the same conditions, were harvested after 140 min incubation with 0.5 mM IPTG and FLAG-SddA levels were analysed by Western blot using α-FLAG antibody (right) and SDS-PAGE followed by Coomassie staining was used as loading control (left). Two specific bands were detected labelled as FL (full length) and D (digested). (C) Quantification of FLAG-SddA FL and D bands in Western blots as shown in (B), normalized using the Coomassie-stained SDS-PAGE. Values are average ± SD of two repeats, * p-value < 0.05 and n.s. not significant. (TIF) [file pgen.1011626.s012.tif]

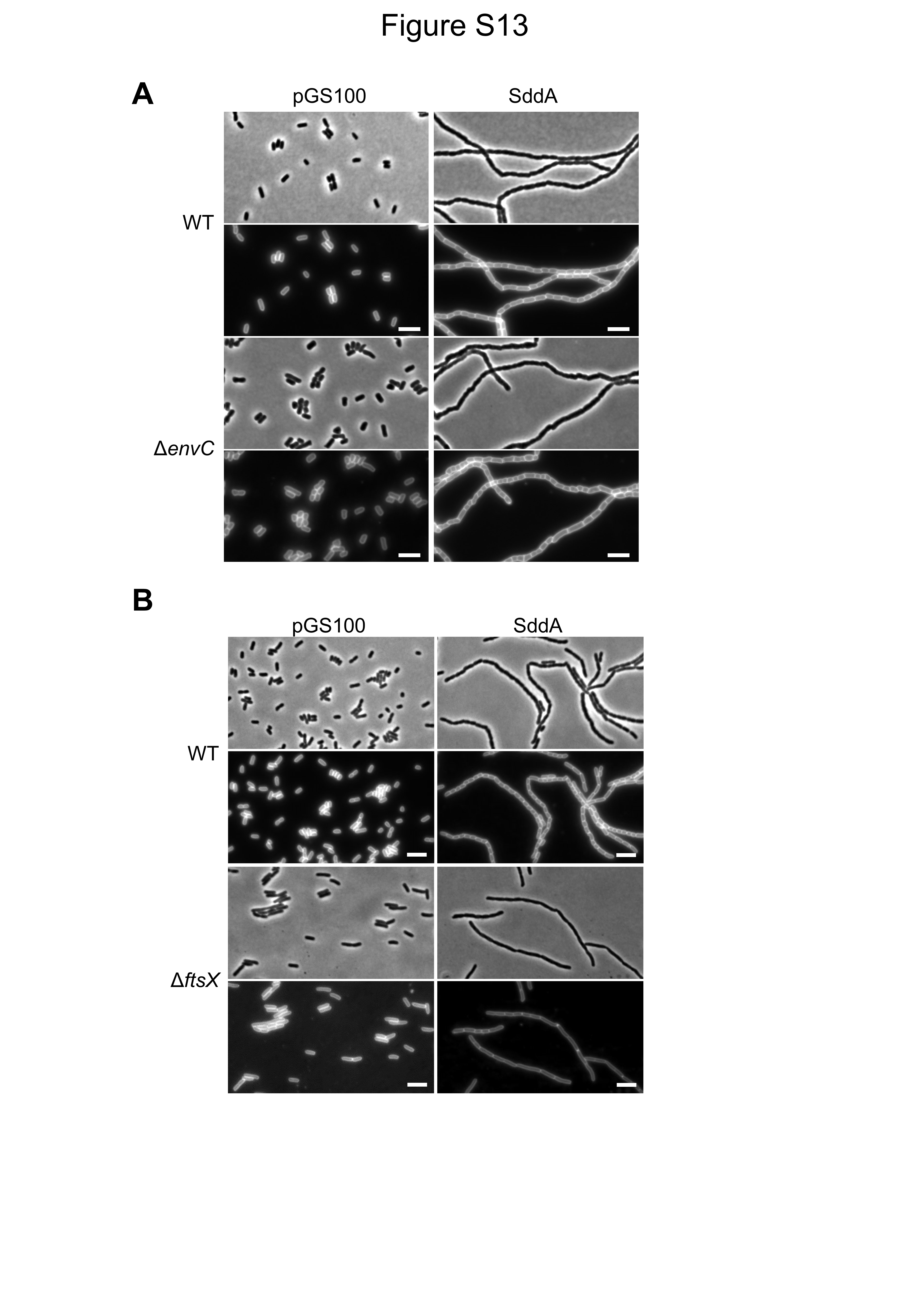

Supplement: S13 Fig — (A) BW25113 (WT) and BW25113 ΔenvC (ΔenvC) cells harbouring pGS100 (ev) or pGS100 expressing sddA (SddA) were grown at 37°C in LB with 20 µg ml-1 chloramphenicol and expression was induced with 0.5 mM IPTG for 140 min. Samples were imaged by phase contrast and fluorescence microscopy (FM5–95). (B) BW25113 (WT) and BW25113 ΔftsX (ΔftsX) cells harbouring pGS100 (ev) or pGS100 expressing sddA (SddA) were grown at 30°C in LB with 20 µg ml-1 chloramphenicol supplemented with 0.2 M sucrose and expression was induced with 0.5 mM IPTG for 230 min. Samples were imaged by phase contrast and fluorescence microscopy (FM5–95). Representative images are shown. Scale bar is 5 µm. (TIF) [file pgen.1011626.s013.tif]

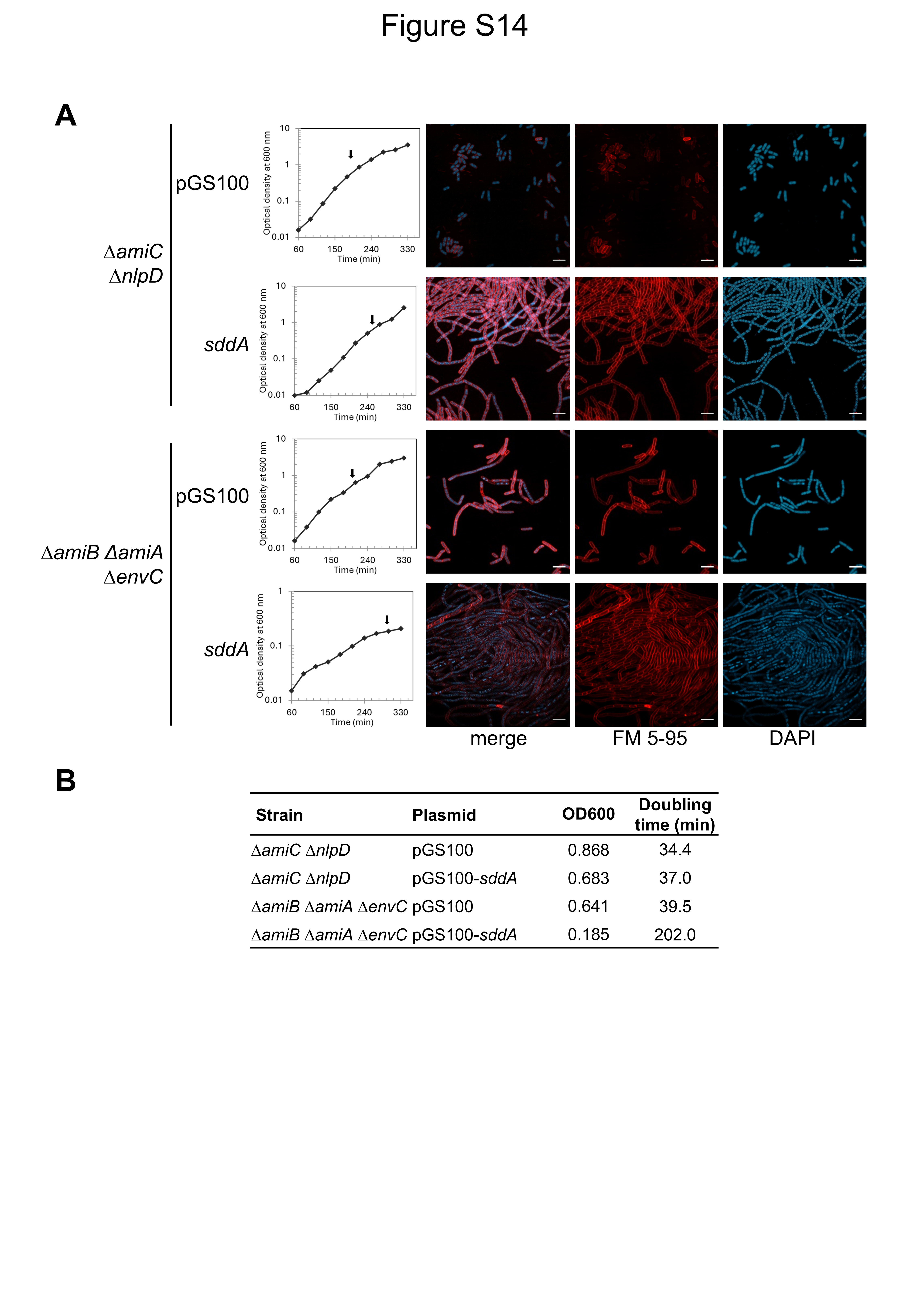

Supplement: S14 Fig — (A) ∆amiC ∆nlpD and ∆amiB ΔamiA ∆envC cells harbouring pGS100 or pGS100 expressing sddA were grown in LB with 5% NaCl supplemented with chloramphenicol at 25 µg ml-1. Samples were collected (arrows) stained with FM5–95 (red, cell membrane) and DAPI (blue, nucleoid), immobilized and imaged by confocal fluorescence microscopy. Representative images are shown. Scale bar is 5 µm. (B) Doubling time of cultures shown in panel A. (TIF) [file pgen.1011626.s014.tif]

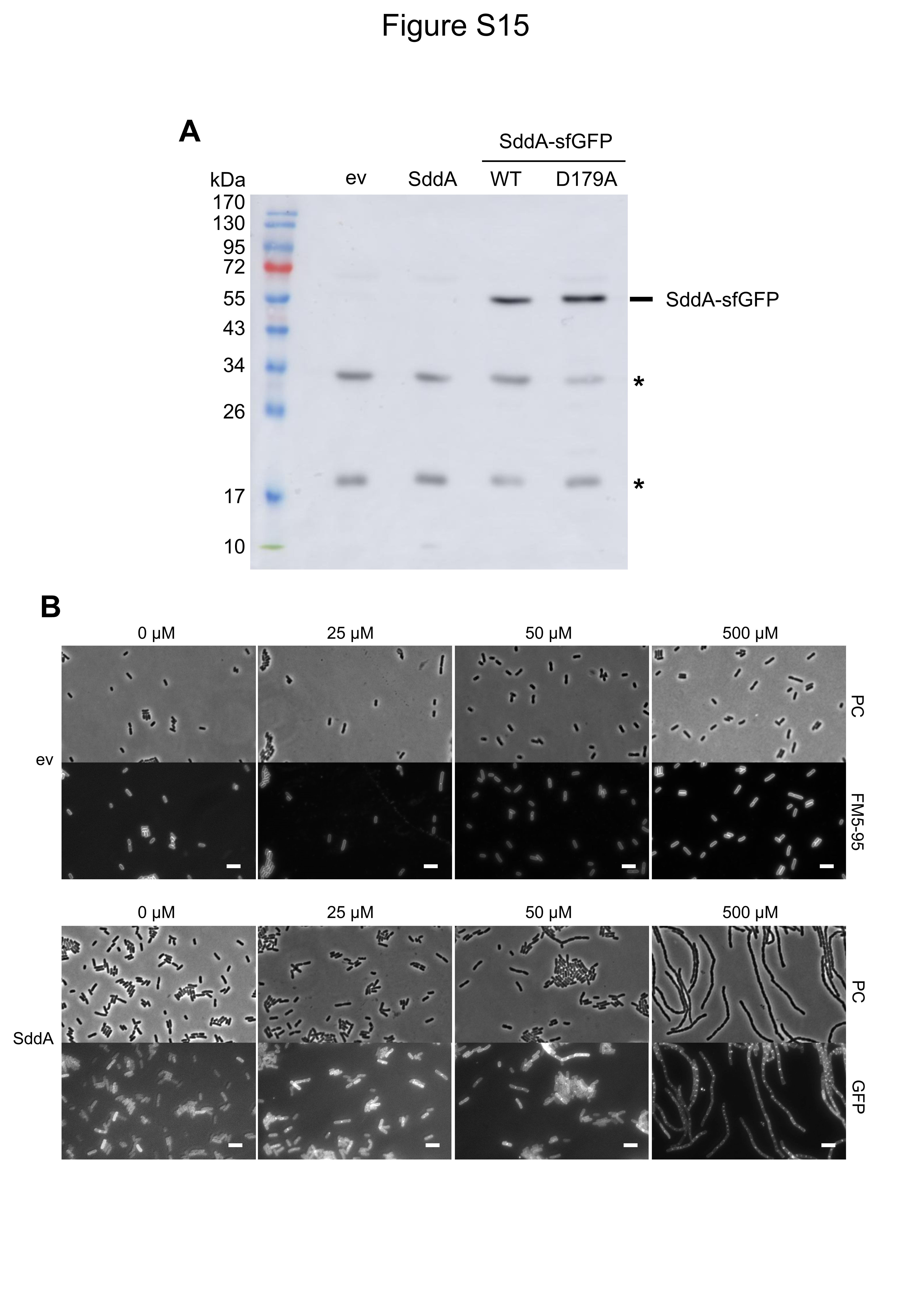

Supplement: S15 Fig — (A) BW25113 cells harbouring pGS100 (ev) or pGS100 expressing sddA, sddA::sfgfp (WT) or sddA D179A::sfgfp (D179A) were grown in LB with 20 µg ml-1 chloramphenicol at 37°C and expression of the fluorescent constructs was induced with 0.5 mM IPTG for 140 min. Samples were pelleted, protein extracts were obtained by sonication and quantified by BCA. 15 µg of each extract was loaded per lane, separated by SDS-PAGE and GFP was immunodetected by Western blot using α-GFP antibody. (*, unspecific bands) (B) BW25113 cells harbouring pGS100 (ev) or pGS100 encoding sddA::sfGFP (SddA) were grown in LB with 20 µg ml-1 chloramphenicol at 37°C and expression of the fluorescent construct was induced with the indicated amount of IPTG for 140 min. For ev, samples cell membranes were stained with FM5–95 dye. Samples were imaged by phase contrast (PC) and fluorescence microscopy (GFP or FM5–95). Representative images are shown. Scale bar is 5 µm. (TIF) [file pgen.1011626.s015.tif]

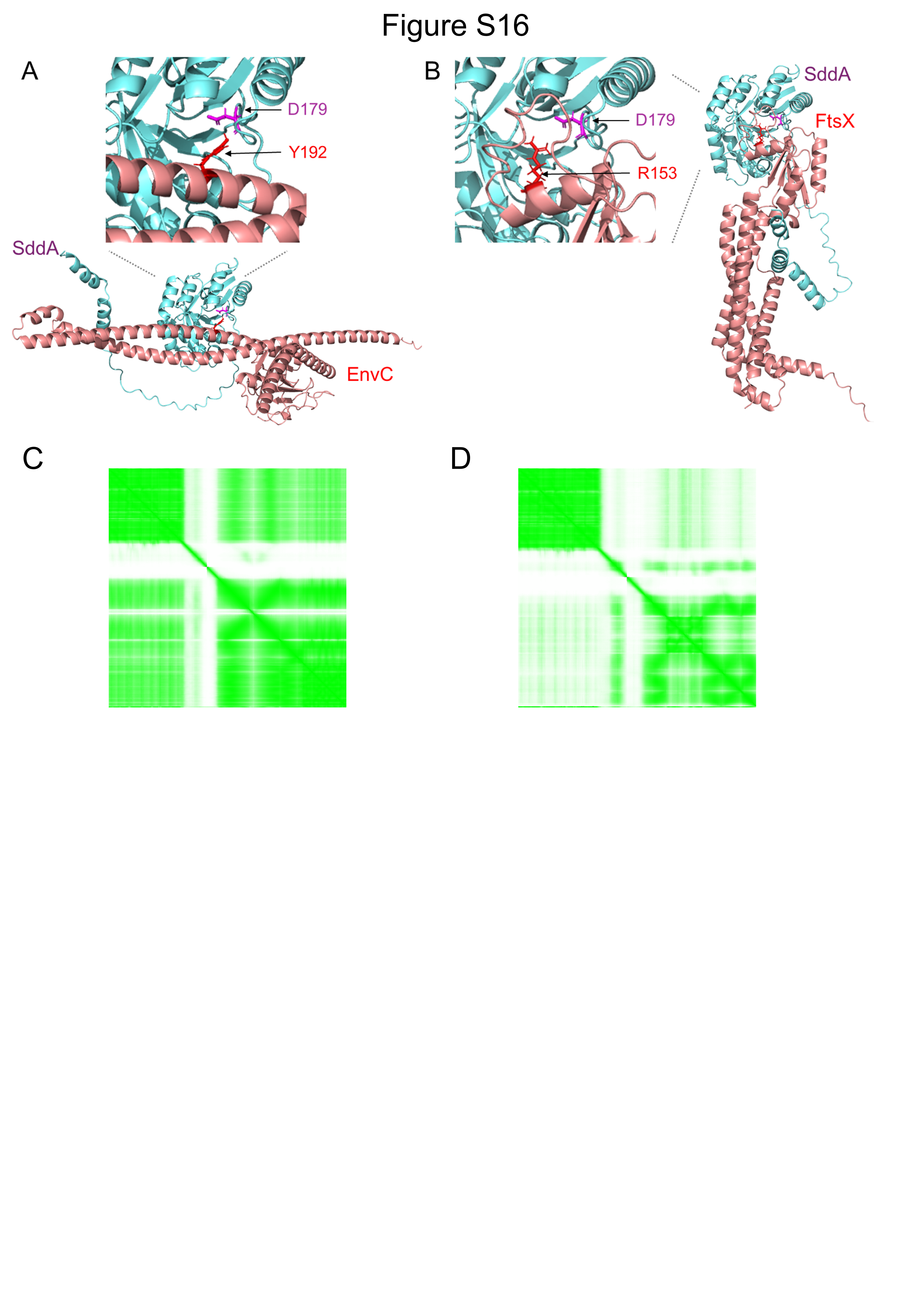

Supplement: S16 Fig — Best models obtained by Alphafold of hypothetical SddA-EnvC, (A), and SddA-FtsX complexes, (B); and their corresponding Predicted Aligned Error (PAE) matrices (C) and (D), respectively. Each cell in the PAE matrices represents the estimate alignment error (in Å) between pairs of residues across the predicted protein structure, with darker shades indicating lower PAE and higher model confidence. (TIF) [file pgen.1011626.s016.tif]

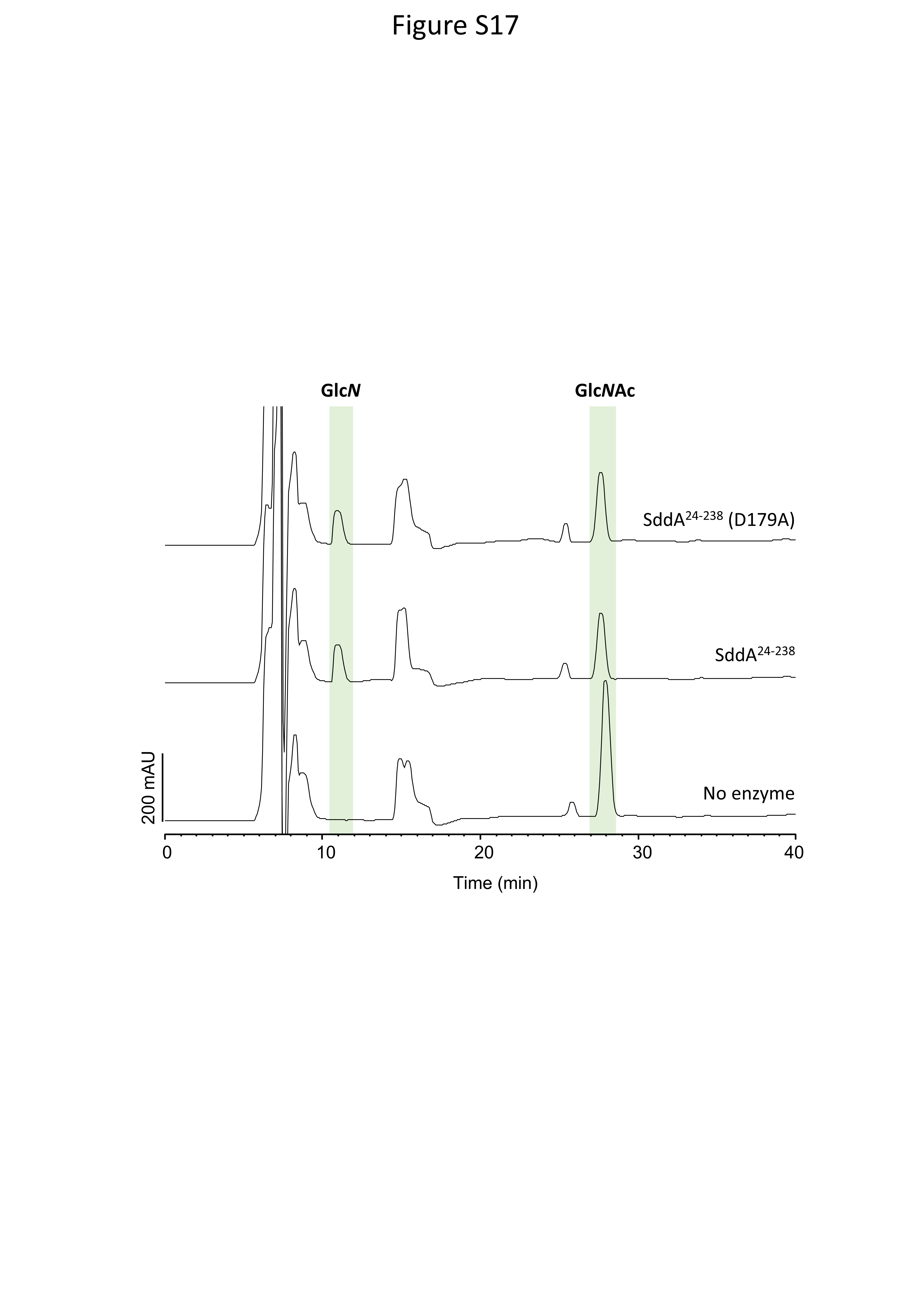

Supplement: S17 Fig — Chromatograms of the analysis of denuded glycan strands treated first with buffer, His-SddA24-238 or His-SddA24-238 D179A, and then with the lytic transglycosylase MltA. MltA fully digested denuded strands producing anhydro sugars. Reactions contained 2 µM of enzyme and were incubated at 37°C for 24 h. Peak labelled GlcN corresponds to GlcN-MurNAcAnh and peak labelled GlcNAc corresponds to GlcNAc-MurNAcAnh. (TIF) [file pgen.1011626.s017.tif]

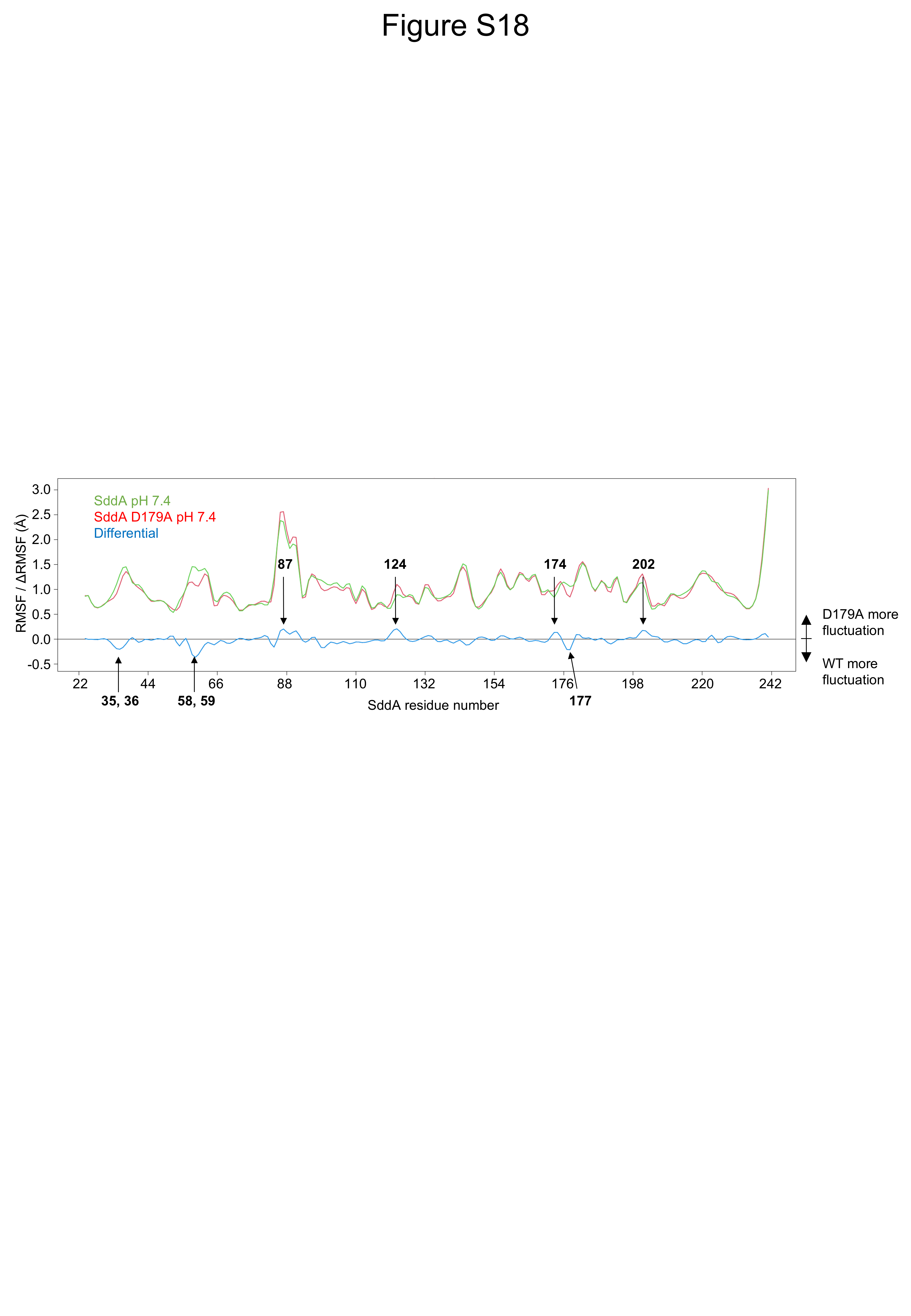

Supplement: S18 Fig — Residue-resolved root mean square fluctuations (RMSF) of wild-type (WT, green) and D179A mutant (red) SddA, calculated from molecular dynamics simulations. The blue curve represents the difference in fluctuations (ΔRMSF) between WT and mutant (WT - mutant). Negative ΔRMSF values indicate greater rigidity in the mutant, while positive values reflect increased flexibility. Residues involved in interaction with EnvC, as predicted by AlphaFold, are highlighted in the analysis. (TIF) [file pgen.1011626.s018.tif]

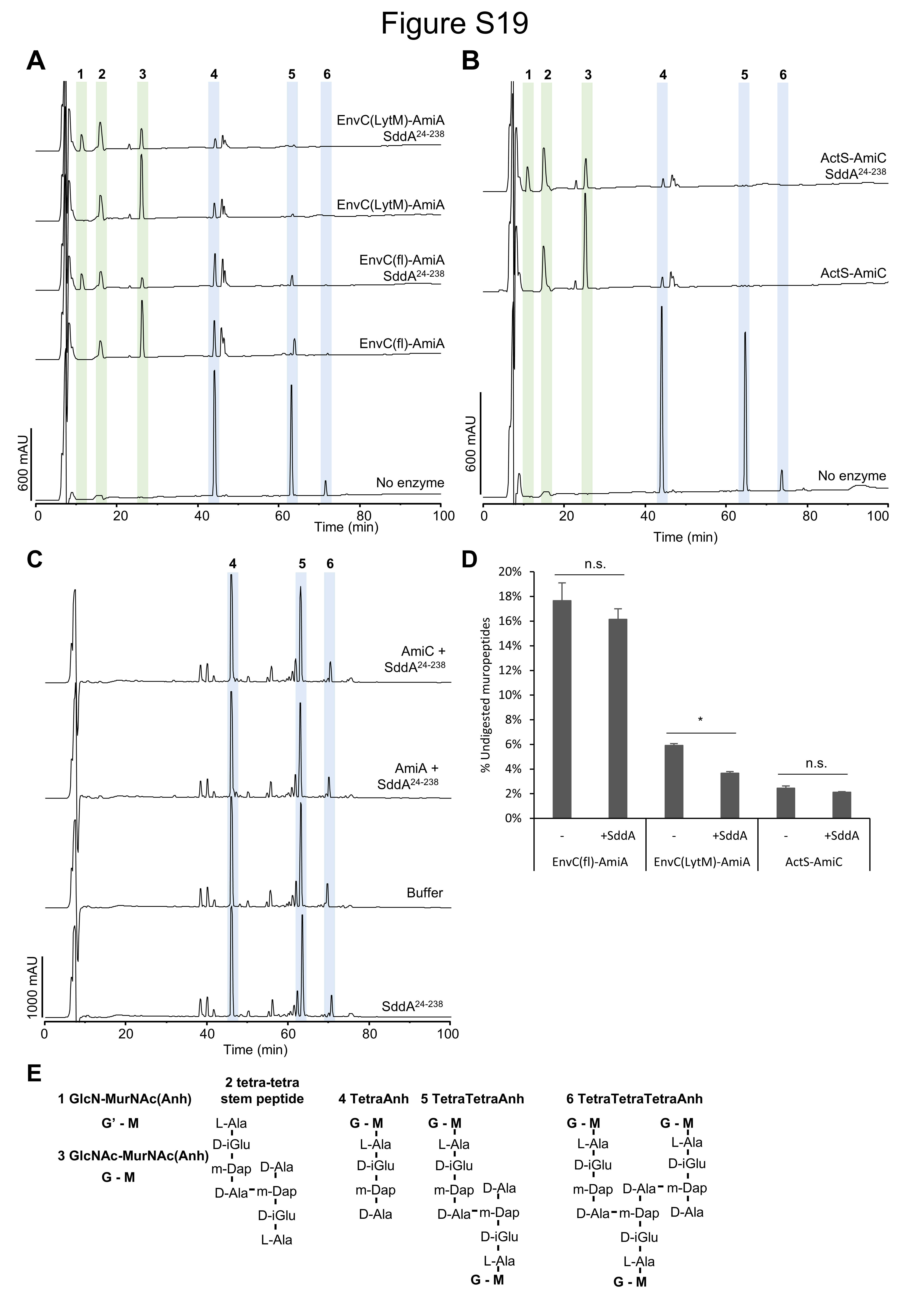

Supplement: S19 Fig — (A, B) HPLC-based end-point activity assays for EnvC-AmiA or ActS-AmiC amidase-activator pairs, in the presence or absence of SddA24-238. Sacculi from E. coli BW25113Δ6LDT were incubated with 2 µM of the indicated amidase and activator, in the presence or absence of 4 µM His-SddA24-238 (SddA24-238). EnvC(fl) indicates the EnvC construct containing the LytM and coiled-coiled domain whereas EnvC(LytM) indicates the construct with only the LytM domain. Reactions were incubated for 2 h (A) or 1 h (B) at 37°C. Representative chromatograms are shown. (C) Chromatograms for the control reactions showing no activation of AmiA or AmiC by SddA24-238. Reactions contained 2 µM of each protein and E. coli MC1061 PG and were incubated for 2 h at 37°C. (D) Quantification of muropeptides peak areas (peaks 4, 5 and 6) in reactions depicted in A and B, normalized against the areas of those peaks in the controls with no enzyme. Values are average + /- variation of 2 reactions. There was a slight activation of EnvC(LytM)-AmiA activity by SddA. (E) Identity and structures of the peaks labelled in A-C and Fig 6A. G stands for GlcNAc, M for MurNAcAnh and G’ for GlcN. (TIF) [file pgen.1011626.s019.tif]
