# Supplementary material for: A novel peptidoglycan deacetylase modulates daughter cell separation in E. coli
Source: PLoS Genet. 2025 Sep 5;21(9):e1011626. doi: 10.1371/journal.pgen.1011626 (PMC12440217; doi:10.1371/journal.pgen.1011626)
Supplement: S1 Table — (DOCX) [file pgen.1011626.s020.docx]

**S1 Table. Strains used in this work.**

| **Strain** | **Relevant features, characteristics** | **Reference/Source** |
| --- | --- | --- |
| DH5alpha | Cloning strain | Thermo |
| BL21(DE3) | Overexpression of His-SddA variants | Sigma |
| BW25113 | *lacI^q^* *rrnB*_T14_ *ΔlacZ*_WJ16_ *hsdR514*  Δ*araBAD*_AH33_ Δ*rhaBAD*_LD78_ | [1] |
| BW25113 Δ*sddA*::kan | BW25113 Δ*sddA(=yibQ)*::kan | This work |
| BW25113 Δ*sddA* | BW25113 Δ*sddA*::FRT | This work |
| BW25113 Δ*actS* Δ*nlpD* | BW25113 Δ*actS*::FRT Δ*nlpD*::FRT | This work |
| BW25113 Δ*actS* Δ*nlpD* Δ*sddA* | BW25113 Δ*actS*::FRT Δ*nlpD*::FRT Δ*sddA*::FRT | This work |
| BW25113 Δ*actS* Δ*envC* | BW25113 Δ*actS*::FRT Δ*envC*::FRT | This work |
| BW25113 Δ*actS* Δ*envC* Δ*sddA* | BW25113 Δ*actS*::FRT Δ(*envC-sddA)*::FRT | This work |
| BW25113 Δ*envC* Δ*nlpD* | BW25113 Δ*envC*::kan Δ*nlpD*::FRT | This work |
| BW25113 Δ*envC* Δ*nlpD* Δ*sddA* | BW25113 Δ*nlpD*::FRT Δ(*envC-sddA)*::kan | This work |
| BW25113 Δ*nlpD* | BW25113 ∆*nlpD*::*aph* | [2] |
| BW25113 Δ*envC* | BW25113 ∆envC::*aph* | [2] |
| MP001 | BW25113 ∆*dedD*::FRT | This work |
| MP005 | BW25113 Δ*sddA* | This work |
| MP006 | BW25113 ∆*dedD*::FRT Δ*sddA* | This work |
| MP055 | BW25113 ∆*ftsX* | This work |
| BW25113 ∆*tolR::frt* | BW25113 ∆*tolR::frt* | [3] |
| BW25113 *tolR D23R* | BW25113 *tolR D23R* | [3] |
| MFDpir | MG1655 RP4-2-Tc::[Mu1::*aac*(3)IV-Δ*aphA*-Δ*nic35*-ΔMu2::*zeo*] Δ*dapA*::(*erm-pir*) Δ*recA* | [4] |
| MPW54 | BW25113 ∆*tolR::frt* ∆*nlpD::aph* | This work |
| MPW55 | BW25113 ∆*tolR::frt* ∆*envC::aph* | This work |
| MPW56 | BW25113 ∆*envC*::aph | This work |
| BW25113 Δ*actS* | BW25113 ∆*actS::frt* | [2] |
| BW25113 Δ*envC* Δ*sddA* | BW25113 ∆*envC::frt* ∆*sddA::frt* | This work |
| BW25113 Δ*actS* Δ*sddA* | BW25113 ∆*actS::frt* ∆*sddA::frt* | This work |
| BW25113 Δ*nlpD* Δ*sddA* | BW25113 ∆*nlpD::frt* ∆*sddA::frt* | This work |
| BW25113 Δ*amiC* Δ*nlpD* | BW25113 *∆amiC::frt* ∆*nlpD::frt* | [2] |
| BW25113 Δ*amiA* Δ*amiB* Δ*envC* | BW25113 ∆*amiA::frt ∆amiB::frt* ∆*envC::frt* | [2] |
| MC1061 | K-12 F– λ– Δ(*ara*-*leu*)7697 [*araD*139]B/r Δ(*codB*-*lacI*)3 *galK*16 *galE*15 e14– *mcrA*0 *relA*1 *rpsL*150(Str R) *spoT*1 *mcrB*1 *hsdR*2(*r^-^m*^+^) | [5] |
| BW25113Δ6LDT | BW25113 Δ*ldtA* Δ*ldtB* Δ*ldtC* Δ*ldtD* Δ*ldtE* Δ*ldtF* | [6] |
| BW27783 | BW25113 Δ(*araFGH*) Φ(Δ*araEp* P_CP8_−*araE*) | [7] |
| BW27783 Δ*prc* | BW27783 Δ*prc*::*frt* | This work |
| AP698 | BW25113 pCH-ss*^dsbA^*-sfGFP-iSPOR | This work |
| AP699 | BW25113 Δ*sddA::FRT* pCH-ss^dsbA^-sfGFP-iSPOR | This work |

**REFERENCES**

1. Datsenko KA, Wanner BL. One-step inactivation of chromosomal genes in *Escherichia coli* K-12 using PCR products. Proc Natl Acad Sci U S A. 2000;97(12):6640-5. doi: 10.1073/pnas.120163297. PubMed PMID: 10829079; PubMed Central PMCID: PMCPMC18686.

2. Gurnani Serrano CK, Winkle M, Martorana AM, Biboy J, More N, Moynihan P, et al. ActS activates peptidoglycan amidases during outer membrane stress in *Escherichia coli*. Mol Microbiol. 2021;116(1):329-42. Epub 20210323. doi: 10.1111/mmi.14712. PubMed PMID: 33660879; PubMed Central PMCID: PMCPMC8360153.

3. Gray AN, Egan AJ, Van't Veer IL, Verheul J, Colavin A, Koumoutsi A, et al. Coordination of peptidoglycan synthesis and outer membrane constriction during *Escherichia coli* cell division. eLife. 2015;4:e07118. Epub 2015/05/08. doi: 10.7554/eLife.07118. PubMed PMID: 25951518; PubMed Central PMCID: PMCPMC4458516.

4. Ferrieres L, Hemery G, Nham T, Guerout AM, Mazel D, Beloin C, et al. Silent mischief: bacteriophage Mu insertions contaminate products of *Escherichia coli* random mutagenesis performed using suicidal transposon delivery plasmids mobilized by broad-host-range RP4 conjugative machinery. J Bacteriol. 2010;192(24):6418-27. Epub 20101008. doi: 10.1128/JB.00621-10. PubMed PMID: 20935093; PubMed Central PMCID: PMCPMC3008518.

5. Casadaban MJ, Cohen SN. Analysis of gene control signals by DNA fusion and cloning in *Escherichia coli*. J Mol Biol. 1980;138(2):179-207. doi: 10.1016/0022-2836(80)90283-1. PubMed PMID: 6997493.

6. Kuru E, Lambert C, Rittichier J, Till R, Ducret A, Derouaux A, et al. Fluorescent D-amino-acids reveal bi-cellular cell wall modifications important for *Bdellovibrio bacteriovorus* predation. Nat Microbiol. 2017;2(12):1648-57. Epub 20171003. doi: 10.1038/s41564-017-0029-y. PubMed PMID: 28974693; PubMed Central PMCID: PMCPMC5705579.

7. Khlebnikov A, Datsenko KA, Skaug T, Wanner BL, Keasling JD. Homogeneous expression of the P(BAD) promoter in *Escherichia coli* by constitutive expression of the low-affinity high-capacity AraE transporter. Microbiology (Reading). 2001;147(Pt 12):3241-7. doi: 10.1099/00221287-147-12-3241. PubMed PMID: 11739756.
