# Supplementary material for: A novel peptidoglycan deacetylase modulates daughter cell separation in E. coli
Source: PLoS Genet. 2025 Sep 5;21(9):e1011626. doi: 10.1371/journal.pgen.1011626 (PMC12440217; doi:10.1371/journal.pgen.1011626)
Supplement: S2 Table — (DOCX) [file pgen.1011626.s021.docx]

**S2 Table. Plasmids used in this work**

| **Plasmid** | **Relevant features, characteristics** | **Reference/Source** |
| --- | --- | --- |
| pET28a-HSddA | pET28a(+) derivative, for overproduction of mature SddA (without signal peptide, residues 24-319) plus an N-terminal His_6_-tag followed by a thrombin cleavage site | This work |
| pET28a-HSddA^24-238^ | pET28a-HSddA with a stop codon introduced at position 238, to overproduce a truncated protein. | This work |
| pET28a-HSddA^24-238^ D31A D32A | pET28a-HSddA^24-238^ with D31A and D32A (corresponding to positions 8 and 9 in the mature protein), introduced by directed mutagenesis | This work |
| pET28a-HSddA^24-238^ H78A | pET28a-HSddA^24-238^ with H78A, introduced by directed mutagenesis | This work |
| pET28a-HSddA^24-238^ H123A | pET28a-HSddA^24-238^ with H123A, introduced by directed mutagenesis | This work |
| pET28a-HSddA^24-238^ D179A | pET28a-HSddA^24-238^ with D179A (corresponding to position 156 in the mature protein), introduced by directed mutagenesis | This work |
| pET28a-His-AmiD | pET28a(+) derivative, for overproduction of soluble AmiD (no signal peptide, AmiD^18-276^) plus an N-terminal His_6_-tag followed by a thrombin cleavage site. | Emily Mulligan |
| pMSS | pJFK118EH derivative. Overexpression of soluble MltA (20-aa removed, and N-terminal Cys changed to Met). | [1] |
| pTB327 | *bla* *lacI^q^* PT7::*h-sumo-amiA* | [2] |
| pTB203 | *bla* *lacI^q^* PT7::*h-sumo-amiC*(32-418) | [2] |
| pET28a-His-EnvC | pET28a derivative; expresses *envC* from the T7 and fused at N-terminal with 6xHis tag. | [3] |
| pTB104 | *bla* *lacI^q^* PT7::*h-sumo-^lyt^envC* | [2] |
| pTB119 | *bla* *lacI^q^* PT7::*h-sumo-nlpD*(27-379) | [2] |
| pET28a His-*actS* | pET28a derivative; expresses *actS* from the T7 promoter starting from amino acid 27 and fused at N-terminal with 6xHis tag. | [3] |
| pGS100 | pGZ119EH derivative, contains TIR sequence downstream of ptac; Cam^R^. | [4] |
| pGS100-*sddA* | pGZ119H derivative; expresses *sddA*(1-319) from the tac promoter; Cam^R^. | This work |
| pGS100-*envC* | pGZ119H derivative; expresses *envC*(1-419) from the tac promoter; Cam^R^. | This work |
| pGS100-*envC-sddA* | pGZ119H derivative; expresses *envC*-*sddA* from the tac promoter; Cam^R^. | This work |
| pGS100-*FLAG-sddA* | pGS100-*sddA* derivative expressing *sddA*(1-24)- DYKDDDDK-*sddA*(25-319); Cam^R^. | This work |
| pGS100-*FLAG-sddA H123A* | pGS100-*sddA* derivative; expresses *sddA*(1-24)- DYKDDDDK-*sddA*(25-319) with H123A; Cam^R^. |  |
| pGS100-*envC-FLAG-sddA* | pGS100-*envC-sddA* derivative; adding DDYKDDDK between residues 24 and 25 in *sddA*; Cam^R^. | This work |
| pBAD24 | pBR derivative; contains the P_BAD_ promoter of the arabinose operon, and its regulatory gene *araC*; Amp^R^ | [5] |
| pBAD24-*nlpD* | pBAD24 derivative; expresses *nlpD*(1-379) from the P_BAD_ promoter; Amp^R^ | This work |
| pBAD24-*envC* | pBAD24 derivative; expresses *envC*(1-419) from the P_BAD_ promoter; AmpR | This work |
| pACBSR | p15A-ori, P_BAD_ promoter, I-*SceI* endonuclease and λ Red genes; Cam^R^ | [6] |
| pAND101 | *B. subtilis* *amyE* integration vector encoding sfGFP | [7] |
| pGEC | *oriT*, R6K-ori, polylinker flanked by two I-SceI restriction sites; Km^R^ | [8] |
| pMP018 | pGEC encoding up-*sddA*-down | This work |
| pMP019 | pGEC encoding up-∆*sddA*-down | This work |
| pMP107 | pGEC encoding up-*ftsX*-down | This work |
| pMP108 | pGS100 encoding *sddA::sfgfp* | This work |
| pMP110 | pGS100 encoding *sddA D179A* | This work |
| pMP112 | pGEC encoding up-*ΔftsX*-down | This work |
| pMP116 | pGS100 encoding *sddA D179A::sfgfp* | This work |
| pCH-ss^dsbA^-sfGFP-iSPOR | P_T5_-lac::^dsbA^ss-*sfGFP*-*ftsN*^SPOR^, Cam^r^ | [9] |

**REFERENCES**

1. Van Straaten KE, Dijkstra BW, Thunnissen AM. Purification, crystallization and preliminary X-ray analysis of the lytic transglycosylase MltA from *Escherichia coli*. Acta Crystallogr D Biol Crystallogr. 2004;60(Pt 4):758-60. Epub 20040323. doi: 10.1107/S0907444904002574. PubMed PMID: 15039577.

2. Uehara T, Parzych KR, Dinh T, Bernhardt TG. Daughter cell separation is controlled by cytokinetic ring-activated cell wall hydrolysis. EMBO J. 2010;29(8):1412-22. Epub 20100318. doi: 10.1038/emboj.2010.36. PubMed PMID: 20300061; PubMed Central PMCID: PMCPMC2868575.

3. Gurnani Serrano CK, Winkle M, Martorana AM, Biboy J, More N, Moynihan P, et al. ActS activates peptidoglycan amidases during outer membrane stress in *Escherichia coli*. Mol Microbiol. 2021;116(1):329-42. Epub 20210323. doi: 10.1111/mmi.14712. PubMed PMID: 33660879; PubMed Central PMCID: PMCPMC8360153.

4. Sperandeo P, Pozzi C, Deho G, Polissi A. Non-essential KDO biosynthesis and new essential cell envelope biogenesis genes in the *Escherichia* coli *yrbG*-*yhbG* locus. Res Microbiol. 2006;157(6):547-58. Epub 20060209. doi: 10.1016/j.resmic.2005.11.014. PubMed PMID: 16765569.

5. Guzman LM, Belin D, Carson MJ, Beckwith J. Tight regulation, modulation, and high-level expression by vectors containing the arabinose P_BAD_ promoter. J Bacteriol. 1995;177(14):4121-30. doi: 10.1128/jb.177.14.4121-4130.1995. PubMed PMID: 7608087; PubMed Central PMCID: PMCPMC177145.

6. Herring CD, Glasner JD, Blattner FR. Gene replacement without selection: regulated suppression of amber mutations in *Escherichia coli*. Gene. 2003;311:153-63. doi: 10.1016/s0378-1119(03)00585-7. PubMed PMID: 12853150.

7. Miguel-Arribas A, Val-Calvo J, Gago-Cordoba C, Izquierdo JM, Abia D, Wu LJ, et al. A novel bipartite antitermination system widespread in conjugative elements of Gram-positive bacteria. Nucleic Acids Res. 2021;49(10):5553-67. doi: 10.1093/nar/gkab360. PubMed PMID: 33999173; PubMed Central PMCID: PMCPMC8191782.

8. Seco EM, Fernandez LA. Efficient markerless integration of genes in the chromosome of probiotic *E. coli* Nissle 1917 by bacterial conjugation. Microb Biotechnol. 2022;15(5):1374-91. Epub 20211109. doi: 10.1111/1751-7915.13967. PubMed PMID: 34755474; PubMed Central PMCID: PMCPMC9049610.

9. Lyu Z, Yahashiri A, Yang X, McCausland JW, Kaus GM, McQuillen R, et al. FtsN maintains active septal cell wall synthesis by forming a processive complex with the septum-specific peptidoglycan synthases in *E. coli*. Nat Commun. 2022;13(1):5751. Epub 20220930. doi: 10.1038/s41467-022-33404-8. PubMed PMID: 36180460; PubMed Central PMCID: PMCPMC9525312.
