# Supplementary material for: A novel peptidoglycan deacetylase modulates daughter cell separation in E. coli
Source: PLoS Genet. 2025 Sep 5;21(9):e1011626. doi: 10.1371/journal.pgen.1011626 (PMC12440217; doi:10.1371/journal.pgen.1011626)
Supplement: S3 Table — (DOCX) [file pgen.1011626.s022.docx]

**S3 Table. Oligonucleotides used in this work**

| **Name** | **Sequence 5’ 🡪 3’** | **Construct / purpose** |
| --- | --- | --- |
| YibQ_FW_NdeI | CATCATCATATGGGCAAACTTGCCATCGTC | pET28a-HYibQ |
| YibQ_REV_XhoI | CATCATCTCGAGCCTTTAATTTGCGCTAGCG | pET28a-HYibQ |
| YibQ_N239stop_FW | CAGCTTGCTGtaaGAACCGCAGG | pET28a-HYibQ^24-238^ |
| YibQ_N239stop_REV | CTGGCTTTCACCAGCGTA | pET28a-HYibQ^24-238^ |
| FW_HYibQ_D31AD32A | CATCGTCATTgctgctTTTGGGTATC | pET28a-HYibQ^24-238^ D31A D32A |
| RV_HYibQ_D31AD32A | GCAAGTTTGCCCATATG | pET28a-HYibQ^24-238^ D31A D32A |
| FW_SDMyibQD179A | GTATTCCTCGcCGATTCGCAAAATG | pET28a-HYibQ^24-238^ D179A, pGS100-*sddA* D179A |
| Rv_SDMyibQD179A | CTTCCGTTTGATCACCTTC | pET28a-HYibQ^24-238^ D179A, pGS100-*sddA* D179A |
| yibQ_NtFLAG_FW | gatgacgacaagAAACTTGCCATCGTCATTGATGATTTTGG | pGS100-FLAG-*sddA*, pGS100-envC-FLAG-*sddA* |
| yibQ_NtFLAG_REV | gtctttgtagtcGCCAGCAAGTACGGGGGA | pGS100-FLAG-sddA, pGS100-envC-FLAG-sddA |
| Fw_yibQ_D31AD32A | CATCGTCATTgctgctTTTGGGTATCG | pGS100-*sddA* D31A D32A |
| Rv_yibQ_D31AD32A | GCAAGTTTGCCAGCAAGT | pGS100-*sddA* D31A D32A |
| sddA_H78A_F | AGTGTTGATTgcgCTCCCGATGGCACCG | pET28a-HYibQ^24-238^ H78A |
| sddA_H78A_R | TCGTGCCCGCTGTTA | pET28a-HYibQ^24-238^ H78A |
| sddA_H123A_F | GATCAACAACgcgATGGGCAGCAAGATGACCTC | pET28a-HYibQ^24-238^ H123A; pGS100-*FLAG*-*sddA* H123A |
| sddA_H123A_R | CCCACGGCATAGGGC | pET28a-HYibQ^24-238^ H123A; pGS100-*FLAG*-*sddA* H123A |
| FwupftsX | TCCATATGCAGCCGGGTG | Δ*ftsX* confirmation |
| RvdownftsX | TCCAGTGAACGGCGAAGG | Δ*ftsX* confirmation |
| FwintftsX | ACGGCGCATTGCAGG | Δ*ftsX* confirmation |
| RvintftsX | AGCAGGCATTCATCG | Δ*ftsX* confirmation |
| FwupYibQseq | AACAGTTGAGCGAGCTGC | Δ*sddA* confirmation |
| RvdownYibQseq | TCAACCGGCGTATTAACC | Δ*sddA* confirmation |
| FwupnlpD | TATTATCGATACCGTGGAGG | Δ*nlpD* confirmation |
| RvintnlpD | TTACCCACCTGCAAGGTCTG | Δ*nlpD* confirmation |
| FwupenvC | CAAACTTTCTGACATCGCGC | Δ*envC* confirmation |
| RvintenvC | TTCAGAAGCACCGATAACC | Δ*envC* confirmation |
| FwintenvC | AGGTTTGCAATCAGGCCCATC | Δ*envC* confirmation |
| RvdownenvC | TAATACAGCGACGGAGATAG | Δ*envC* confirmation |
| AP863/yibQext-fw | GATTGCACTGGTGGGCAGC | Δ*sddA* confirmation |
| AP864/yibQext-rv | CAATGTGCGCAGCGTGAAG | Δ*sddA* confirmation |
| FwpGECLIC | ACTAGTCCTAGGGCATGC | pMP018, pMP107 |
| RvpGECLIC | ATATGGAGCTCACGCGTC | pMP018, pMP107 |
| FwupYibQLIC | GTCTCGAGACGCGTGAGCTCCATATAGAAGCACGTGAGGC | pMP018 |
| RvdownYibQLIC | TCCCGGTGCATGCCCTAGGACTAGTTTTATCGCCGTGATG | pMP018 |
| FwYibQSDM | CTAGCGCAAATTAAAGGC | pMP019 |
| RvYibQSDM | AACTTATCTTCCCAACCAC | pMP019 |
| FwupFtsXLIC | GTCTCGAGACGCGTGAGCTCCATATAAGCTGATCTGTGGG | pMP107 |
| RvdownFtsXLIC | TCCCGGTGCATGCCCTAGGACTAGTCCGATGTTACCTTCC | pMP107 |
| FwpGS100LIC | TGACTTGGCTGTTTTGGCGG | pMP108 |
| RvyibQLIC | ATTTGCGCTAGCGTTAAACTTC | pMP108 |
| FwyibQsfGFPLIC | GGCGAAGTTTAACGCTAGCGCAAATCGTAAAGGCGAAGAG | pMP108 |
| RvsfGFPLIC | TCA TCC GCC AAA ACA GCC AAG TCA TTT GTA CAG TTC ATC C | pMP108 |
|  |  |  |
| FwSDMyibQD179A | GTA TTC CTC GcC GAT TCG CAA AAT G | pMP110 |
| RvSDMyibQD179A | CTT CCG TTT GAT CAC CTT C | pMP110 |
| FwdelftsXSDM | TAA AAG CGT GTT ATA CTC TTT CC | pMP112 |
| RvdelftsXSDM | TTA TTC ATG GCC CAC GCC | pMP112 |
| AP907/*yibQ_*EcoRI_fw | CCG GAA TTC ACC TTG TTT CCA TTT CGT CGT AAC | pGS100-*sddA* |
| AP908/*yibQ_*HindIII_rv | CCC AAG CTT TTA TTA ATT TGC GCT AGC GTT AAA C | pGS100-*sddA*; pGS100-*envCsddA* |
| AP937/*envC_*EcoRI_fw | CCG GAA TTC ACC ATG ACA CGG GCC GTG AAA CC | pGS100-*envC*; pGS100-*envCsddA* |
| AP938/*envC_*HindIII_rv | CCC AAG CTT TTA TTA TCT TCC CAA CCA CGG C | pGS100-*envC* |
| AP909/Δ*envC*Δ*sddA_*fw | AAG AGA TGA CTG GTA AGC CGC TGT TCA TCG TGG AAT AAT CCC TCC CCA TGT GTA GGC TGG AGC TGC TTC G | Δ*envC* Δ*sddA* |
| AP910/Δ*envC*Δ*sddA_*rv | GTC GCA CCA TCG CAT TAC GCG TAG CGC CTT TAA TTT GCG CTA GCG TTA AAC ATA TGA ATA TCC TCC TTA | Δ*envC* Δ*sddA* |
| AP970/NlpD_EcoRI_fw | CAG GAG GAA TTC ACC ATG AGC GCG GGA AGC CCA AAA TTC | pBAD24_*nlpD* |
| AP998/NlpD_HindIII_rev | TCT GAA GCT TTT ATC GCT GCG GCA AAT AAC | pBAD24_*nlpD* |
|  |  |  |
|  |  |  |
